# Enzymology of Pyran Ring A Formation in Salinomycin Biosynthesis

**DOI:** 10.1002/anie.201507090

**Published:** 2015-09-17

**Authors:** Hanna Luhavaya, Marcio V B Dias, Simon R Williams, Hui Hong, Luciana G de Oliveira, Peter F Leadlay

**Affiliations:** Department of Biochemistry, University of Cambridge 80 Tennis Court Road, Cambridge CB2 1GA (UK) E-mail: pfl10@cam.ac.uk; Department of Microbiology, Institute of Biomedical Science, University of São Paulo, Av. Prof. Lineu Prestes 1374, 05508-000, São Paulo-SP (Brazil); University Chemical Laboratory, University of Cambridge Lensfield Road, Cambridge CB2 1EW (UK); Department of Organic Chemistry, University of Campinas UNICAMP, Cidade Universitária Zeferino Vaz s/n P.O. Box 6154, 13083-970, Campinas-SP (Brazil)

**Keywords:** biosynthesis, cyclases, dehydratases, polyethers, polyketide synthases

## Abstract

Tetrahydropyran rings are a common feature of complex polyketide natural products, but much remains to be learned about the enzymology of their formation. The enzyme SalBIII from the salinomycin biosynthetic pathway resembles other polyether epoxide hydrolases/cyclases of the MonB family, but SalBIII plays no role in the conventional cascade of ring opening/closing. Mutation in the *salBIII* gene gave a metabolite in which ring A is not formed. Using this metabolite in vitro as a substrate analogue, SalBIII has been shown to form pyran ring A. We have determined the X-ray crystal structure of SalBIII, and structure-guided mutagenesis of putative active-site residues has identified Asp38 and Asp104 as an essential catalytic dyad. The demonstrated pyran synthase activity of SalBIII further extends the impressive catalytic versatility of α+β barrel fold proteins.

Tetrahydropyran rings are widespread features of complex reduced polyketide natural products, including compounds of outstanding potential or utility as agrochemicals or pharmaceuticals, such as the coccidiostat and anticancer ionophore salinomycin[1] (Figure 1). Such compounds are formed on bacterial modular type I polyketide synthases (PKSs), giant multimodular enzymes that use a remarkable assembly-line logic for biosynthesis of diverse bioactive natural products.[2] Each module catalyzes a specific cycle of polyketide chain elongation, and contains a ketosynthase (KS) domain that condenses activated acyl and malonyl units, an acyltransferase (AT) that specifies the type of extender unit introduced, and an acyl carrier protein (ACP) that tethers the growing polyketide chain while it is processed by optional ketoreductase (KR), dehydratase (DH), and enoylreductase (ER) domains.[3] Some assembly-line PKSs (*trans*-AT PKSs) lack module-specific AT domains and show considerable variation in their domain order compared to *cis*-AT PKSs.[4] PKSs use further enzymes to introduce additional chemical diversity[2a, 5] and there is great interest in identifying and characterizing these auxiliary enzymes.

**Figure 1:**
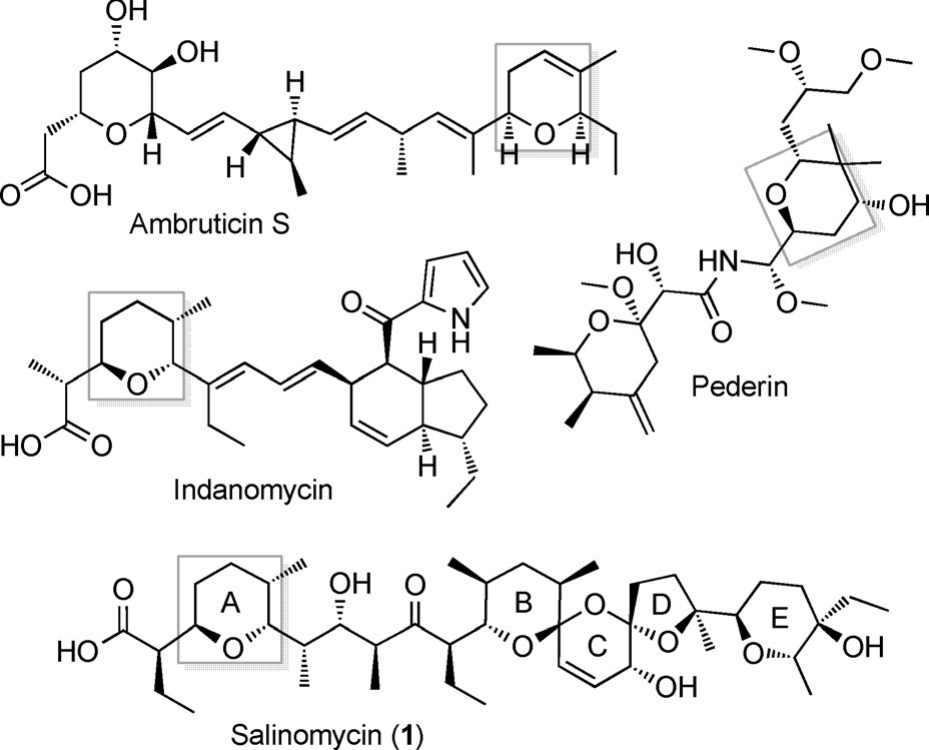
Structures of selected polyketides containing pyran rings (highlighted in gray).

In polyether ionophores, tetrahydrofuran and tetrahydropyran rings are formed by flavin-linked oxidation of diene- or triene-containing intermediates to epoxides,[6] and their subsequent ring-opening catalyzed by epoxide hydrolases/cyclases of the MonB family.[7] However, gene cluster analysis has suggested that other pyran rings are formed by other routes.[8] In *trans*-AT PKS systems, such as the one that produces pederin (Figure 1), it has been shown that certain modules contain a DH-like pyran synthase (PS) domain that acts on the α,β-unsaturated thioester product of the adjacent DH domain to produce the pyran, before the polyketide chain is passed to the downstream module.[8c,d,e,f] PS domains are not found in *cis*-AT PKSs, but recent work on the DH domain of module 3 of the PKS for ambruticin (Figure 1) showed that this domain has dual activity, first dehydrating and then catalyzing stereoselective ring formation.[8g] Sequence comparisons reveal no clear differences between AmbDH3 and monofunctional DH domains from either *cis*- or *trans*-AT PKSs, so the basis of the dual activity is unknown. The *cis*-AT PKS for indanomycin (Figure 1) contains an integral domain (Cyc11) which has been proposed[8h] from gene cluster analysis to form the pyran at the western end of the molecule. However, experimental evidence for this is lacking.

The salinomycin biosynthetic gene cluster[9] encodes a *cis*-AT PKS predicted to give rise to an enzyme-bound diene that is oxidized by epoxidase SalC to a diepoxide. This in turn is ring-opened by tandem action of SalBI and SalBII, from the MonB-family of epoxide hydrolases/cyclases. The *sal* PKS shows several unusual features, including a *cis*-double bond at C18–C19 inserted by the novel dehydratase[9c,d] (and putative spirocyclase[9d]) SalE, and the lack of a conventional off-loading enzyme.[9] Notably, the final extension module of the PKS contains neither a DH domain nor a pederin-like PS domain, so the origin of the pyran ring at the western end of the molecule has been obscure. The gene *salBIII*[9a] encodes a third member of the MonB-family of epoxide hydrolases/cyclases (Supporting Information, Figure S10), and we wished to test the hypothesis that this enzyme catalyzes pyran formation. Previous analysis of a mutant deleted in the *salE* dehydratase gene has shown that the metabolites **5**, **6**, and **7** that accumulate (Supporting Information, Figure S15) all have pyran ring A present,[9c,d] as does diene **3** (Figure 2), from a mutant lacking the epoxidase SalC.[9a] These results imply that pyran formation is an early post-PKS event.

**Figure 2:**
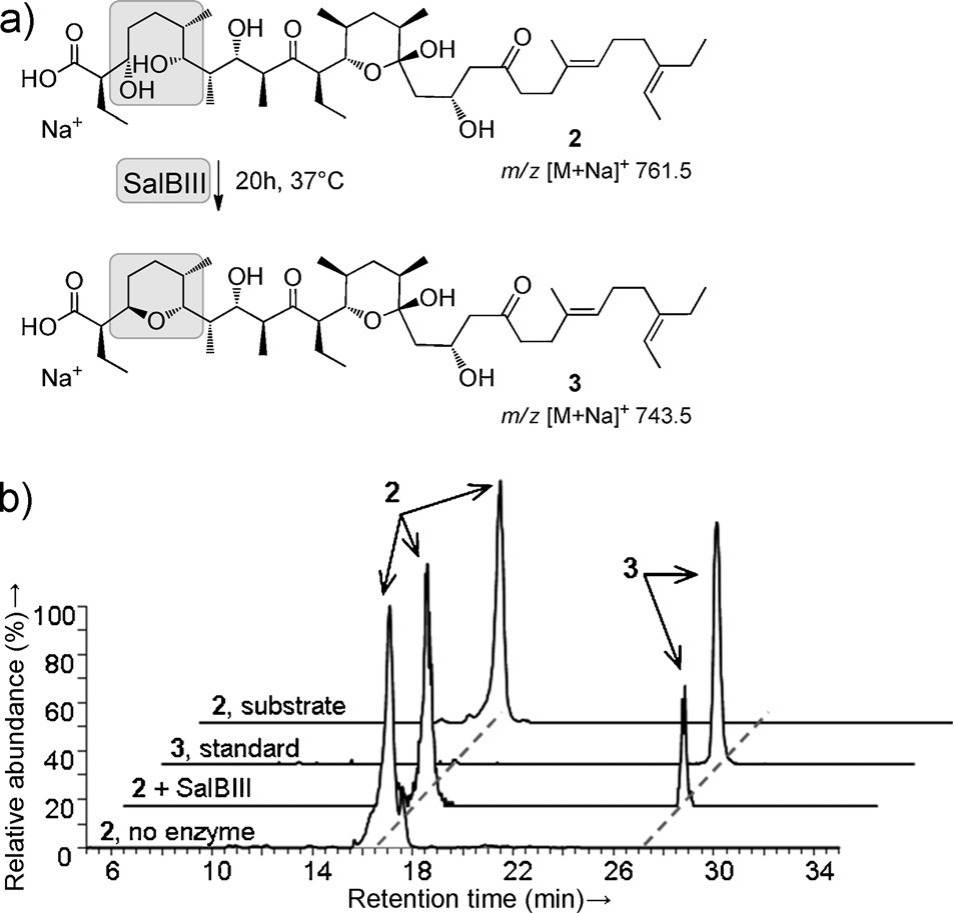
In vitro analysis of the reaction catalyzed by recombinant SalBIII. a) Reaction catalyzed by SalBIII. b) Stacked spectra from HPLC-MS analysis showing formation of 3 in the presence of SalBIII.

First, we fully confirmed the configuration of metabolite **3** isolated from the Δ*salC* mutant (Supporting Information, Figure S29) by using 1D- and 2D-NMR analysis (Supporting Information, Table S1, Figures S1–S9, S33–S46). Next, we deleted the *salBIII* gene in *Streptomyces albus*. LC-MS analysis of extracts from the Δ*salBIII* mutant strain showed that salinomycin (*m*/*z* 773.5) production was abolished (Figure S17). Instead, two metabolites with *m*/*z* 791.5 were observed at retention times 8.6 and 11.1 min, respectively. Production of salinomycin was fully restored in the *S.* *albus* Δ*salBIII* mutant when complemented in *trans* with the wild-type *salBIII* gene. Their MS–MS fragmentation pattern suggests that these metabolites represent *bis*-epoxide shunt products in which pyran ring A is not yet formed (Figures S18, S19). To further clarify the role of SalBIII in pyran formation, we constructed a double mutant lacking both *salBIII* and the epoxidase *salC*. LC-MS analysis of extracts from the Δ*salC*/Δ*salBIII* mutant strain showed loss of **3** and the appearance of small amounts of a new metabolite **2** with *m*/*z* 761.5 (Figure 2; Supporting Information, Figure S21). Complementation in *trans* with a wild-type copy of SalBIII restored the production of **3** (Figure S21). Gene cluster analysis[9a,c] was used to deduce the stereochemistry of **2**, and the chemical structure was experimentally confirmed by MS–MS analysis (Figures S22–S28). Comparison of **2** and **3** (Figure 2) strongly implicated SalBIII in converting a PKS-bound form of **2**, tethered in thioester linkage to an ACP domain, into a PKS-bound form of **3**. This could conceivably occur either by direct S_N_2-type displacement, or by acting first as a dehydratase to form an α,β-unsaturated thioester, and then as a cyclase catalyzing oxa-conjugate addition.

To confirm the activity of SalBIII, we reconstituted pyran formation in vitro using recombinant SalBIII enzyme purified from *Escherichia coli* (Figure S30) with **2** as a surrogate for the natural ACP-tethered substrate. Metabolite **2** was indeed slowly converted into **3** by purified SalBIII (Figure 2; Supporting Information, Figure S31). The identity of the product of the in vitro reaction was confirmed by showing that the MS–MS spectrum was identical to that of authentic **3** (Figure S32). The assumed α,β-unsaturated acid intermediate of the dehydration–addition pathway was not detected, so if this mechanism is followed then either pyran ring formation outpaces dehydration or the two reactions are tightly coupled without release of the intermediate.

To provide a structural framework for understanding the activity of SalBIII, we have solved the X-ray crystal structure of SalBIII at 1.8 Å resolution. The statistics for data collection, refinement, and validation are presented in the Supporting Information (Table S10). Phases were determined by molecular replacement. There are two protomers in the asymmetric unit, consistent with the fact that SalBIII is a homodimer in solution (Figure S30). Each subunit adopts the cone-shaped α+β barrel fold[10] first recognized in the structure of scytalone dehydratase[10a] in which three or four α-helices pack against the concave side of a curved five- or six-stranded mixed β-pleated sheet, creating a largely hydrophobic active site cavity (Figure 3), closed at one end. This fold has subsequently been observed in diverse enzymes utilizing general acid–general base catalysis.[10] The most similar 3D structures to that of SalBIII are those of the MonB-type epoxide hydrolases,[7] but sequence alignment (Figure S10) shows that in SalBIII one of the two catalytic residues (Asp38 and Glu65 in Lsd19/LasB N-terminal domain) is missing, and overall its sequence diverges significantly from other members of this family (Figure S11). Using the crystal structure of SalBIII as a guide (Figure 3), eight residues were identified as contributing to its active site: Tyr14, Asp38, Arg45, Tyr54, Asn58, Ile65, Asp104, and Trp121. Site-specific mutagenesis was used to mutate these residues, and the mutant genes were tested for their ability to restore salinomycin production to the *S.* *albus* Δ*salBIII* strain. The results (summarized in Table S9) showed that each of the Tyr14Phe, Tyr54Phe, Asn58Ala, Ile65Thr, and Arg45Ala mutants substantially restored salinomycin production, while only low salinomycin production was seen for the Trp121Ala mutant. In contrast, alteration of either Asp38 or Asp104 (to either Ala or Asn) gave wholly inactive mutants. These residues are suitably placed in the active site to operate as a catalytic dyad, to accomplish both dehydration and oxa-conjugate addition. Their positioning on the same side of the active site pocket appears to rule out a direct displacement mechanism in which one group acts as a general acid to assist the departing C3-OH and the other as a general base to assist the C7-OH nucleophile. The mechanism we propose (Scheme 1) involves an E1cb-like elimination, in which general base-catalyzed cleavage of a C–H bond at C2 to form a stabilized enolate is followed by β-elimination from C3.[11] Asp38 is proposed to furnish the general base for proton abstraction, and the formation and stabilization of the enolate may also be favored by general acid catalysis involving polarization of the thioester carbonyl. Asp104 is proposed to assist β-elimination from C3. The α,β-unsaturated thioester then undergoes oxa-conjugate addition of the C7 hydroxy group, assisted by Asp104, leading to *syn* addition across the double bond. Asp38 may assist ring formation by hydrogen-bonding to the thioester carbonyl, or may provide the proton at C2 of the pyran product. Further experiments to test this mechanistic proposal are in progress.

**Figure 3:**
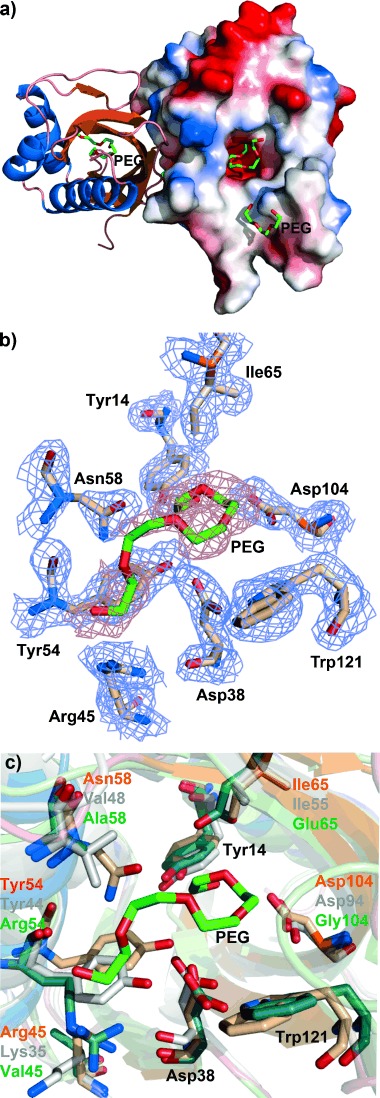
Crystal structure of SalBIII. a) Overall fold of SalBIII. The right-hand monomer is shown with its electrostatic surface (blue: negatively charged; red: positively charged). PEG: polyethylene glycol. b) Predicted active site residues in SalBIII. c) Superposition of the active sites of SalBIII (orange), Cyc11 (model, gray) and Lsd19 (green).

**Scheme 1:**
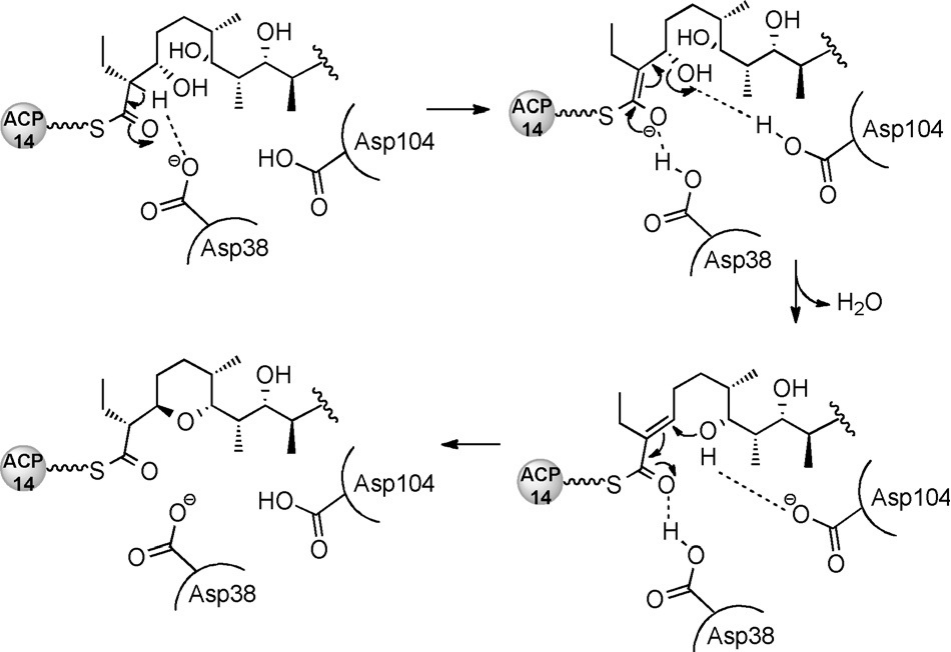
Proposed mechanism of the intramolecular cyclization catalyzed by SalBIII. Asp38 and Asp104 constitute a catalytic dyad. ACP_14_: the terminal acyl carrier protein domain of the *sal* PKS.

Sequence alignment of the putative cyclase domain Cyc11 from the indanomycin PKS[8h] with SalBIII (Figure S12) revealed that Cyc11 contains equivalent residues (Asp28 and Asp94) to those found here to be essential to SalBIII activity. Superimposition of the modeled active site structure of Cyc11 and that of Lsd19/LasB C-terminal domain[10e] onto the active site of SalBIII shows that they occupy exactly equivalent positions (Figure 3). Also, Arg45, Tyr54, and Ile65 of SalBIII have close counterparts in Cyc11, consistent with one or more of these residues also playing a catalytic role (Figure 3). It therefore seems probable that Cyc11 adopts a similar catalytic mechanism for pyran formation. The identification of SalBIII as a pyran synthase provides an additional tool for potential chemoenzymatic synthesis of pyrans, and further extends the impressive catalytic versatility shown by proteins and domains adopting the α+β barrel fold.

## Supporting Information

As a service to our authors and readers, this journal provides supporting information supplied by the authors. Such materials are peer reviewed and may be re-organized for online delivery, but are not copy-edited or typeset. Technical support issues arising from supporting information (other than missing files) should be addressed to the authors.

miscellaneous_information
