# Supplementary material for: Enzymology of Pyran Ring A Formation in Salinomycin Biosynthesis
Source: Angew Chem Int Ed Engl. 2015 Sep 17;54(46):13622–5. doi: 10.1002/anie.201507090 (PMC4648038; doi:10.1002/anie.201507090)
Supplement: Supplementary file 1 — miscellaneous_information [file anie0054-13622-sd1.pdf]

Angewandte Chemie
Eine Zeitschrift der Gesellschaft Deutscher Chemiker

Supporting Information

Enzymology of Pyran Ring A Formation in Salinomycin Biosynthesis

*Hanna Luhavaya, Marcio V. B. Dias, Simon R. Williams, Hui Hong, Luciana G. de Oliveira, and Peter F. Leadlay**

anie_201507090_sm_miscellaneous_information.pdf

Table of Contents

1. Supplementary Methods 3

1.1 General analytical procedures 3

1.2 Materials, DNA isolation and manipulation 4

1.3 Bacterial strains and culture conditions 4

1.4 General strategy for vector construction for in-frame gene deletion 5

1.5 Expression and purification of recombinant proteins 5

1.6 Enzyme activity assay 6

1.7 Culture extraction for HPLC-MS analysis of metabolites 6

1.8 Purification of *S. albus* Δ*salC* metabolite **3** 6

1.9 H/D-exchange in small organic molecules 7

1.10 Crystallization and X-ray data collection 7

1.11 Structure determination and refinement 7

2. NMR Analysis of Compound 3 8

3. Supplementary Tables 12

Table S1. NMR data for **3** 12

Table S2. List and description of vectors used 13

Table S3. List and description of strains used 13

Table S4. Oligonucleotide primers used 14

Table S5. HRMS data for **3** ($[M+Na]^+$ and $[M+NH_4]^+$ ions). 15

Table S6. HRMS and HRMS/MS data for compound **2** ($[M+Na]^+$ ion) 15

Table S7. $HRMS^3$ data for compound **2** ($[M+Na]^+$ ion, 761.5→383.2) 15

Table S8. HRMS and HRMS/MS data for compound **B-11** ($[M+Na]^+$ ion). 15

Table S9. Restoration of salinomycin production in *S. albus* Δ*salBIII* by wild type and mutant forms of SalBIII 16

Table S10. Statistics of data collection, refinement and validation of SalBIII 17

4. Supplementary Figures 18

Figure S1. 2D structure determined for **3** 8

Figure S2. NOE correlations used to determine the geometry of the two alkenes in **3** 8

Figure S3. Conformation of the A ring 9

Figure S4. Conformation and coupling constants about the C7-C8 bond 9

Figure S5. Conformation of the B ring 10

Figure S6. Conformation about the C12-C13 bond 10

Figure S7. Conformation about the C18-C19 bond 11

Figure S8. Conformation of the C16-C19 region 11

Figure S9. Structure of **3** 11

Figure S10. Multiple sequence alignment of the polyether epoxide hydrolases, limonene epoxide hydrolase (LEH) and SalBIII 18

Figure S11. Phylogenetic analysis of polyether epoxide hydrolases. 19

Figure S12. Sequence alignment of SalBIII and the Cyc11 domain from indanomycin 19

Figure S13. Schematic illustration of the in-frame deletion of the *salBIII* gene in the salinomycin biosynthetic gene cluster 20

Figure S14. PCR analysis of in-frame deletion of *salBIII* gene in the salinomycin biosynthetic gene cluster in *S. albus* WT and *S. albus* Δ*salC* strains 20

Figure S15. Chemical structures of the metabolites from *S. albus* Δ*salE* mutant 21

Figure S16. Proposed general structure for the metabolites observed in *S. albus* Δ*salBIII* mutant 21

Figure S17. HPLC-MS analysis of the metabolite profile of *S. albus* Δ*salBIII* mutant 22

Figure S18. MS/MS spectra of the molecular ion with m/z 791.5 at 8.6 and 11.1 min from *S. albus* Δ*salBIII* mutant metabolite profile 23

Figure S19. Proposed MS/MS fragmentation pattern and HRMS/MS spectrum of m/z 791.5, compound **B-11** produced by *S. albus* Δ*salBIII*. 25

Figure S20. MS spectrum of deuterated $[M+Na]^+$ ions for compound **B-11** 24

Figure S21. HPLC-MS analysis of the metabolite profile of *S. albus* Δ*salC*/Δ*salBIII* and *S. albus* Δ*salC* mutants 24

Figure S22. HRMS/MS spectrum of m/z 761.5, compound **2** produced by *S. albus* Δ*salC*/Δ*salBIII*. 25

Figure S23. $HRMS^3$ spectrum of m/z 761.5→383.2 from compound **2** produced by *S. albus* Δ*salC*/Δ*salBIII* 25

Figure S24. Proposed fragmentation pathways for compound **2** 26

Figure S25. Proposed fragmentation pathways for compound **2** 27

Figure S26. Comparison of MS spectrum of non-deuterated and deuterated $[M+Na]^+$ ions for **2** 27

Figure S27. MS/MS spectrum of deuterated $[M+Na]^+$ ion of compound **2** 28

Figure S28. MS^3 spectrum of deuterated $[M+Na]^+$ ion 767.5→388.5 of compound **2** 28

Figure S29. HPLC-HRMS analysis of purified compound **3** 29

Figure S30. Analysis of the purified recombinant SalBIII protein. 29

Figure S31. HPLC-MS analysis of conversion of **2** to **3** 30

Figure S32. MS/MS analysis of the molecular ion $[M+Na]^+$ 743.5 corresponding to the assay product and the **3** standard. 30

Figure S33. ^1H NMR spectrum of **3** with assignments 31

Figure S34. ^{13}C NMR spectrum of **3** with assignments 32

Figure S35. DEPT 135 NMR spectrum of **3** 33

Figure S36. DEPT 90 NMR spectrum of **3** 33

Figure S37. DEPT 45 NMR spectrum of **3** 33

Figure S38. ^{13}C NMR spectrum of **3** 33

Figure S39. DQF-COSY NMR spectrum of **3** 34

Figure S40. Edited-HSQC NMR spectrum of **3** 34

Figure S41. HMBC NMR spectrum of **3** 35

Figure S42. NOESY NMR spectrum of **3** 35

Figure S43. HSQC-TOCSY NMR spectrum of **3** 36

Figure S44. TOCSY NMR spectrum of **3** 36

Figure S45. HSQC-HECADE NMR spectrum of **3** 37

Figure S46. G-BIRD-HSQMBC NMR spectrum of **3** 37

5. References 38

6. Main Text Complete References 38

1. Supplementary Methods

1.1 General analytical procedures

NMR data were collected in CD_3CN using Bruker Avance spectrometers using either a 500 DCH cryoprobe operating at 500.05 MHz for ^{1}H and 125.7 MHz for ^{13}C (^{13}C, TOCSY) a 500 TCI cryoprobe operating at 500.13 MHz for ^{1}H and 125.8 MHz for ^{13}C (^{1}H, DEPT, HSQC-TOCSY, HSQC-HECADE, G-BIRD-HSQMBC) or a 700 TCI cryoprobe operating at 700.03 MHz for ^{1}H and 176.02 MHz for ^{13}C (^{1}H, DQF-COSY, HSQC, HMBC, NOESY), in the Chemistry Department, University of Cambridge. Chemical shifts were recorded using an internal deuterium lock for ^{13}C and residual ^{1}H in CD_3CN (δ_H 1.94, δ_C 118.26) and are given in ppm on a scale relative to $\delta_{TMS} = 0$. NMR spectra were processed using Bruker Topspin (v. 3.2). DQF-COSY spectra were acquired with 4k data points in F_2 and 512 increments with 2 scans per increment. TOCSY spectra were acquired using DIPSI2 modulation and a mixing time of 120 ms, 8k data points were acquired in F_2 and 360 increments with 4 scans per increment. The edited HSQC spectra were optimized for 145 Hz with 2k data points in F_2, 512 increments and 4 scans per increment. HMBC spectra optimized for 10 Hz with a three-fold low pass J filter to suppress one bond couplings, 4k data points were acquired in F_2 with 768 increments and 8 scans per increment. Edited-HSQC-TOCSY spectra were optimized for 145 Hz using DIPSI2 modulation and a mixing time of 120 ms, 8k data points were acquired in F_2, 360 increments and 16 scans per increment. NOESY spectra were recorded using a mixing time of 0.6 s, 8k data points in F_2 with 768 increments and 4 scans per increment. The HSQC-HECADE spectrum was recorded with 8k data points in F_2, 512 increments and 36 scans per increment. The J (scale) factor was 1, the TOCSY mixing time 120 ms and the spectrum was optimised for $^1J_{C\text{-}H} = 145$ Hz. The G-BIRD-HSQMBC experiment was recorded with 8K data points in F_2, 480 increments with 40 scans per increment, a delay time $\tau/2$ of 1/8J and optimised for $^1J_{C\text{-}H} = 145$ Hz. All data were zero filled to 1K or 2K in F_1 for processing.

HPLC-MS analysis was performed using an HPLC (Hewlett Packard, Agilent Technologies 1100 series) coupled to a Finnigan MAT LTQ mass spectrometer fitted with an electrospray ionization (ESI) source. HPLC-MS data were processed and deconvoluted using Xcalibur (v. 1.1) (Thermo Finnigan).

For analysis of small molecules the HPLC was fitted with a Prodigy 5µ C18 column (250 mm × 4.6 mm, Phenomenex) column. A solvent system of methanol and water both containing 0.1% formic acid (v/v) was used. Samples were eluted with a linear gradient of 85 to 100% of methanol over 20 min, then 100% methanol over 10 min at a flow rate 0.7 mL min^{-1} (method A). Alternatively a linear gradient of 85 to 100% of methanol over 15 min, then 100% methanol over 8 min at a flow rate 1 mL min^{-1} was used (method B). The mass spectrometer was run in positive ionization mode, scanning from m/z 150 to 1800, and the collision energy was set to 15%. ESI high resolution MS (ESI-HRMS) was carried out on a Thermo Fisher Orbitrap with 60,000 resolution and normalized collision energy of 15%.

For protein mass determination the HPLC was fitted with a Jupiter C4 (250 mm x 2 mm, 5 µm, Phenomenex) column. A solvent system of acetonitrile and water both containing 0.1% (v/v) trifluoroacetic acid was used. Samples were eluted with a linear gradient of 5 to 35% of acetonitrile over 10 min, then from 35 to 95% of acetonitrile over 15 min at a flow rate 0.3 mL min^{-1}.

Analytical protein gel-filtration chromatography was performed on ÄKTApurifier™ UPC 10 (GE Healthcare) fitted with a Superdex® 200 5/150 column (150 mm × 5 mm, 13 µm, GE Healthcare) in Phosphate buffer (50 mM NaH_2PO_4, 150 mM NaCl, pH 7.2) at a flow rate of 0.15 mL min^{-1} over 35 min.

Preparative HPLC purification was performed using a Luna C18(2) column (250 mm x 21.2 mm, 10 µm, Phenomenex) connected to the Agilent 1200 series HPLC. Samples were eluted at a flow rate of 15 mL min^{-1} using the following method: water and acetonitrile as solvents with a gradient of 90 to 95% acetonitrile over 10 min, then 95 to 100% acetonitrile over 20 min, followed by 100% acetonitrile wash for 15 min. Fractions were collected at 0.9 min intervals.

Concentrations of DNA and protein samples were measured using a Nanodrop spectrophotometer (ND-1000 v3.8.1).

1.2 Materials, DNA isolation and manipulation

Plasmids, strains and oligonucleotides (Invitrogen) used in this work are summarized in Tables S2, S3 and S4, respectively.

Restriction endonucleases, Calf intestinal alkaline phosphatase, Phusion® high-fidelity PCR master mix with GC buffer (for cloning), and Gibson assembly® master mix were purchased from New England Biolabs (NEB). Biomix™ Red PCR master mix (for screening purposes) was purchased from Bioline. T4 DNA ligase and fast digest restriction endonucleases were supplied by Fermentas. Lysozyme powder was purchased from Amresco®. Proteinase K powder was manufactured by Melford Laboratories Ltd. All chemicals were from Sigma-Aldrich. All organic solvents used were HPLC grade.

Plasmid DNA was isolated from an overnight culture using the E.Z.N.A.® Plasmid Mini Kit I (Omega Bio-Tek) according to the manufacturer's protocol. High molecular weight genomic DNA from *Streptomyces* strains was isolated using the salting out procedure.[1] Purification of DNA fragments from agarose gels was performed using the KeyPrep Gel DNA Clean Up Kit (Anachem) according to the manufacturer's instructions. Site-directed mutagenesis was performed according to modified QuikChange® site-directed mutagenesis kit (Stratagene) guidelines. DNA sequencing was carried out by the DNA Sequencing Facility in the Department of Biochemistry, University of Cambridge.

1.3 Bacterial strains and culture conditions

Streptomyces albus strains were grown in TSBY liquid medium (3% tryptone soy broth, 10.3% sucrose, 0.5% yeast extract) for isolation of genomic DNA, and on SFM solid medium (2% mannitol, 2% soya flour, 2% agar) for conjugation and strain maintenance. For liquid cultures, the strains were

grown at 30°C with shaking at 220 rpm in a rotary incubator for 36-44 h. For solid culture, the strains were grown at 30°C for 10-12 days.

For salinomycin production, seed medium (sucrose 4%, soybean flour 1%, brewer's yeast 0.5%, $CaCO_3$ 0.2%, pH 7.5) was inoculated with *S. albus* spore suspension and cultured for 36-48 h. 10% inocula of seed culture were used for fermentation medium (soybean flour 1%, starch 0.5%, $CaCO_3$ 0.5%, $(NH_4)_2SO_4$ 0.3%, NaCl 0.2%, $MgSO_4$ 0.01%, KH_2PO_4 0.02%, pH 7.5, after autoclaving sunflower oil was added at final level 6%). Fermentation was carried out at 30°C and 240 rpm in a rotary incubator for 7 days.

E. coli strains were grown on solid (2% agar) or liquid LB (1% tryptone, 0.5% yeast extract, 1% NaCl) or 2TY (1.6% tryptone, 1% yeast extract, 0.5% NaCl) media supplemented with appropriate antibiotics (ampicillin 100 µg mL^{-1}, apramycin 50 µg mL^{-1}, chloramphenicol 25 µg mL^{-1}, kanamycin 50 µg mL^{-1}) at 37°C unless otherwise stated. Liquid cultures were shaken in either conical flasks or Falcon tubes at 180 to 250 rpm during incubation.

1.4 General strategy for vector construction for in-frame gene deletion

Recombinant plasmids for in-frame gene deletions were constructed by ligation of two PCR-amplified DNA fragments (about 2 kb) from the upstream and downstream flanks of the target gene into pYH7[2] vector digested with *Nde*I (Figure S13). The Gibson assembly method was used to perform a three-piece ligation.[3]

Recombinant plasmids were introduced by conjugation into *S. albus* DSM 41398. The donor strain was *E. coli* ET12657/pUZ8002, and conjugation was carried out as described by Luhavaya *et al*.[4] Potential mutants were checked by PCR analysis (Figure S14).

The complementation plasmid pIB-*salBIII* was constructed based upon integrative vector pIB139[5] placing *salBIII* under the *ermE** promoter.

1.5 Expression and purification of recombinant proteins

The expression plasmid pET-SalBIII_C (Table S2) was introduced into *E. coli* BL21(DE3). A single colony was inoculated into 5 mL of LB medium containing 50 µg mL^{-1} kanamycin and grown overnight at 37°C, 250 rpm. Typically 0.6 L of LB medium containing kanamycin in a 2 L flask was inoculated with 6 mL of overnight culture. The culture was incubated at 37°C, 220 rpm until the A_{600} reached 0.6–0.8, when gene expression was induced by adding 1 M isopropyl-β-D-thiogalactopyranoside (IPTG) aqueous solution to a final concentration of 0.2 mM. The culture was further incubated at 20°C, 180 rpm for 16 h. Cells were harvested by centrifugation at 12,000 x *g*, 5 min, 4°C. The cell pellet was resuspended in Binding buffer (40 mM Tris, 0.1 M NaCl, 20 mM Imidazole, pH 7.4) and lysed by sonication. The sonicate was centrifuged at 35,000 x *g*, 45 min, 4°C, after which the soluble fraction was removed, filter sterilized, and subjected to column chromatography.

Ni-NTA His•Bind® Resin (Novagen) column (1 mL bed volume) was pre-charged with nickel ions and equilibrated with Binding buffer. After the sonicate was loaded onto the column, the resin was

washed sequentially with five column volumes of Binding buffer and five column volumes of Washing buffer 1 (40 mM Tris, 0.1 M NaCl, 40 mM Imidazole, pH 7.4), followed by protein elution with three column volumes of Elution buffer (40 mM Tris, 0.1 M NaCl, 300 mM Imidazole, pH 7.4). To remove residual proteins the column was treated with Washing buffer 2 (40 mM Tris, 0.1 M NaCl, 500 mM Imidazole, pH 7.4).

Buffer exchange in protein-containing fractions into Storage buffer (25 mM HEPES, 0.1 M NaCl, pH 7.4) was done by using a PD-10 desalting column (GE Healthcare Life Sciences) according to the manufacturer's protocol. Freshly purified enzyme was immediately used for activity assays, the rest was flash-frozen in liquid nitrogen and stored at -80°C.

The protein yield of SalBIII is 45 mg L^{-1}. The purified proteins were examined by pre-cast NuPAGE® Novex® 4 – 12% Bis-Tris gels with NuPAGE® MES SDS running buffer (Thermo Scientific™) and HPLC-MS (Figure S30).

1.6 Enzyme activity assay

Activity assays for SalBIII were carried out in a total reaction volume of 100 µL containing 2 µL of compound **2** (dissolved in DMSO, concentration unknown) and SalBIII at a final concentration of 50 µM. Reaction mixtures were incubated at 37°C for 20 h in a Storage buffer with pH 6.8. The assay mixture was extracted with ethyl acetate (3 x 400 µL). After evaporation of the solvent, the samples were resuspended in 50 µL of methanol and analyzed by HPLC-MS. Reaction mixture without enzyme added served as a control.

Substrate for the assay (compound **2**) was purified using analytical C18 column because of the low production yield of the metabolite (Section 1.1, HPLC method A). After 24 injections (dried ethyl acetate extract from 0.5 L of culture redissolved in 1.3 mL of methanol) and manual collections, the amount of compound **2** was still below quantification level, but enough for the MS detection.

1.7 Culture extraction for HPLC-MS analysis of metabolites

500 µL samples of culture broth of *S. albus* strains were extracted with 500 µL of ethyl acetate. The solvent was evaporated, the residue was redissolved in 500 µL of methanol and the mixture centrifuged before being subjected to HPLC-MS analysis. 5 µL of the solution was injected.

1.8 Purification of *S. albus* Δ*salC* metabolite 3

A 7-day-old 1.7 L culture broth of *S. albus* DSM 41398 Δ*salC* mutant[6] strain was extracted twice with 2 L of ethyl acetate. The combined organic layers were dried over $MgSO_4$ and the solvent was evaporated, yielding 6.7 g of an oily residue. The latter was redissolved in 0.5 L of hexane and extracted three times with 0.5 L methanol/water (4:1, v/v) mixture. Methanol/water fractions were combined, and methanol was removed by evaporation at reduced pressure. The remaining water layer was extracted three times with 500 mL of ethyl acetate. The combined extracts were dried with anhydrous $MgSO_4$ and the solvent was removed *in vacuo*. 5.5 g of oily residue was obtained. To remove the remaining oil, the sample was further purified by flash chromatography on a column of

silica gel (60 µm particle size, 20 cm × 3 cm). The column was washed with 300 mL of 1:1 (v/v) mixture of hexane/ethyl acetate, and compounds were eluted with 2 L of ethyl acetate/methanol (19:1, v/v). Combined fractions contained 1.6 g of a mixture containing compound **3**. Further purification was achieved by repeated rounds of preparative HPLC. Final fractions were combined and desalted using Chromabond C18 EC column (Macherey-Nagel) yielding around 5.2 mg of **3** (Figure S29). All stages of purification were monitored by direct injection into the Finnigan MAT LTQ mass spectrometer or by HPLC-MS analysis.

1.9 H/D-exchange in small organic molecules

Residual solvent from the sample was removed under the flow of nitrogen. The sample was redissolved in deuterated methanol and incubated at room temperature for 24 h. Then the sample was used for direct injection into the Finnigan MAT LTQ mass spectrometer (Figures S20, S26).

1.10 Crystallization and X-ray data collection

For protein crystallization SalBIII was purified and stored in 50 mM Tris, 200 mM NaCl, pH 7.8 at a final concentration of 20 mg mL^{-1}. Approximately 500 conditions from different commercialized crystallization kits (crystal screen and SaltRx from Hampton Research, JSCG+ and PACT suite from Qiagen, Precipitant Synergy from Jena Bioscience and Wizard I and II from Emerald BioSystem) were screened using a HoneyBee crystallization robot. The volume of protein sample and crystallization solution was 0.4 µL, which was dispensed in sitting drop plates (maintained at 18°C). The best rod shaped crystals could be reproduced using a hanging drop method in Limbro plates and they appeared after 2-4 days in a crystallization condition composed by 25% PEG400, 20% PEG3350, 0.1 mM $MgCl_2$ and 0.1 mM Tris pH 8.0. Crystals for SalBIII were prior cryo-cooled using as a cryo-mixture composed of 20% of PEG400 and 80% of well crystallization condition. X-ray diffraction data were collected at the P13 station on PETRAIII, Hamburg, Germany using a wavelength of 0.977Å and a PILATUS 6M pixel detector. The images were integrated using XDS[7] in the space group $I2_12_12_1$ and at a resolution of 1.8Å. The statistics of data collection and refinement are detailed in Table S10.

1.11 Structure determination and refinement

The structure of SalBIII was solved by molecular replacement using the methods implemented in the program Phaser[8] from the suite CCP4[9]. The structure of the C-terminal domain of Lsd19 (PDB code: 3RGA) was used as a search model.[10] The refinement was carried out using the program REFMAC5[11] or Phenix.refine[12] from Phenix suite[13] version 1.8.4-1496. The structure was manually rebuilt and visualized using the program COOT 0.7.2.[14] The structure was validated using the program MolProbity.[15] Visual analysis was also performed using the program COOT 0.7.2[14] and the figures were prepared using the program PyMol (Schrödinger, LLC). Data collection, refinement and validation statistics are presented in Table S10. The SalBIII atomic coordinates and structure factor have been deposited in the Protein Data Bank, PDB code: 5CXO.

2. NMR Analysis of Compound 3

Inspection of the ^{13}C, DEPT and ^{1}H spectra indicated the presence of two ketones, one carboxylic acid derivative, two trisubstituted double bonds and an acetal. TOCSY and HSQC-TOCSY spectra allowed the identification of seven spin systems (shown below in bold), the connectivity of these was then elucidated using COSY data and the location of the two rings by HMBC correlations. While no TOCSY correlation could be established between H8 and H9 a weak COSY coupling and several HMBC correlations allowed spins systems 1 and 2 to be connected. HMBC correlations then connected the remaining spin systems along with the six unprotonated carbons to give the flat structure (Figure S1). This contains the C1-C16 portion exactly as found in salinomycin and in the salinomycin analogues[4] (Figure S15), a hemiacetal at C17, alcohol at C19, ketone at C21 and two trisubstituted double bonds at C24/C25 and C28/C29.

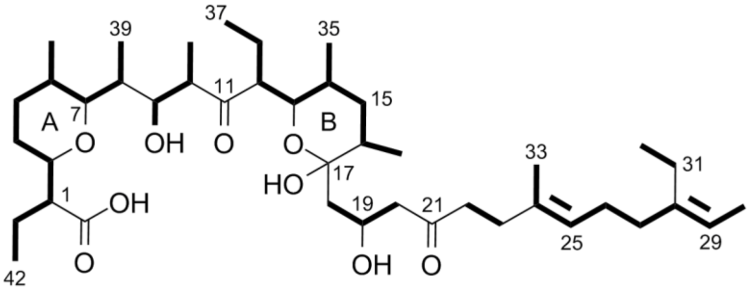

Figure S1. 2D structure determined for **3**. Spin systems as determined by TOCSY shown in bold.

Both double bonds were determined to be *trans* using NOESY data as shown in Figure S2 with $^{3}J_{C-H}$ coupling constants being used to confirm the geometry of the C28-C29 alkene.

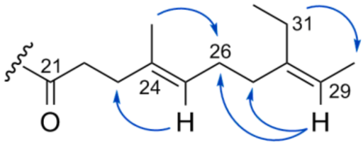

Figure S2. NOE correlations used to determine the geometry of the two alkenes. *(Note. Due to overlap of the H26 and H31 signals the NOE correlations from this signal to H29 and H30 cannot be unambiguously assigned as being due to H26 and H31, respectively. However, an NOE correlation between H27/H29 along with the observed ^{1}H-^{13}C couplings (H29/C27 ^{3}J = 5 Hz and H29/C31 ^{3}J = 8.2 Hz) all agree with a trans configuration. It therefore seems reasonable to assume that the ambiguous correlations are indeed those shown in the figure).*

The structure of the A ring was determined using NOE and ^{1}H-^{1}H coupling constant data. The key NOE correlations were those between H2, H5a and H7 that placed all these on one face and C4a and Me40 that placed these on the other. The configuration of the C2 stereocentre was harder to confirm; the large (10.8 Hz) coupling between H2 and H3 placed these in an antiperiplanar arrangement precluding the use of J-based configurational analysis which cannot distinguish between the two possible diastereomers in this case. No useful NOEs were observed between the

A ring and H41 either. Comparison of the ^1H-^1H coupling constant and NOE correlation data in this region with that for natural salinomycin (**1**) showed a very good match however suggesting the C2 stereocentre be assigned as *R* configured (Figure S3).

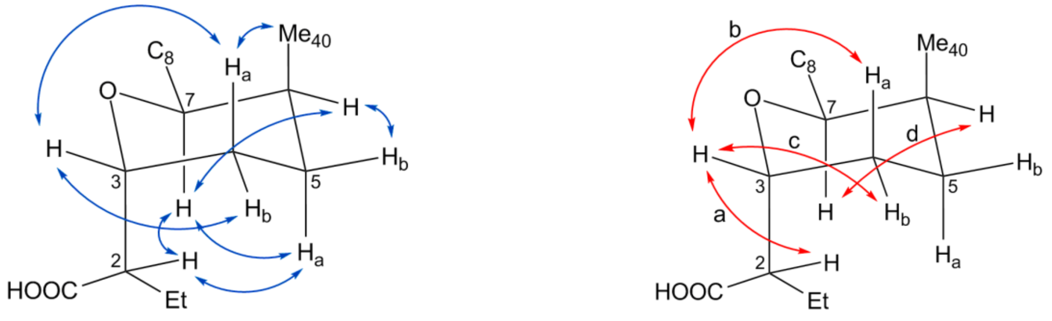

Coupling	$^3J_{H-H}$ in 3, Hz	$^3J_{H-H}$ in 1, Hz
a) H2-H3	10.8	11.0
b) H3-H4a[a]	5.1	4.7
c) H3-H4b[a]	0[b]	0[b]
d) H6-H7	2.4	2.2

[a]Assigments of H3-H4 coupings are arbitrary, H3 shows couplings to H2 of 10.8Hz and 5.1Hz (to 4a or 4b) the small coupling constants for 4a and 4b could not be determined. [b]Too small to determine.

Figure S3. Conformation of the A ring. Key NOE correlations shown in blue and coupling constants in red.

The stereochemistry in the C7-C10 region was assigned using Muratas's J-based configurational analysis method.[16] The required ^1H-^{13}C coupling constants were obtained from HSQC-HECADE[17] and G-BIRD-HSQMBC[18] experiments, the latter being necessary to overcome the small scalar coupling between H8 and H9.

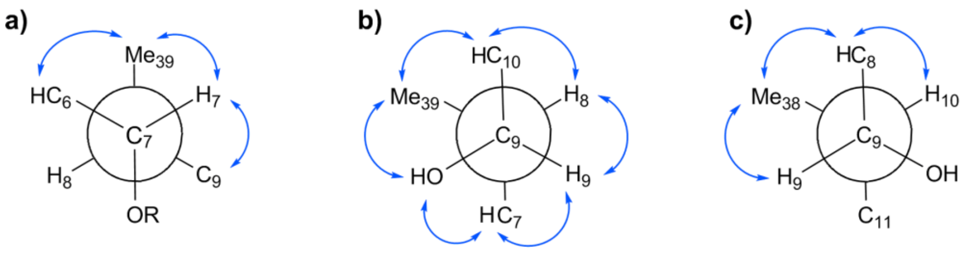

Coupling	J (Hz)	Large/ Small		Coupling	J (Hz)	Large / Small		Coupling	J (Hz)	Large / Small
H7-H8	10.1	Large		H8-H9	*c.* 1.0	Small		H9-H10	10.0	Large
H7-C9	1.8	Small		H8-C9	2.4	Small		H9-C11	nd	*Small*
H7-Me39	1.2	Small		H8-C10	3.4	Small		H9-Me38	0.9	Small
H8-C7	7.0	Large		H9-C7	3.7	Small		H10-C8	2.6	Small
H8-C6	3.1	Small		H9-Me39	6.8	Large		H10-C9	5.7	Large

Expected value for undetermined (nd) coupling constant is shown in *italics*.

Figure S4. a) Conformation and coupling constants about the C7-C8 bond, data fits a B3 conformation. **b)** Conformation and coupling constants about the C8-C9 bond, data fits an A1 conformation. **c)** Conformation and coupling constants about the C9-C10 bond, data a B3 conformation. NOE correlations shown in blue.

H15b showed three large (*c.* 11.6 Hz) couplings indicating that both H14 and H16 were axially oriented and thus Me34 and Me35 equatorial. The H13/H14 coupling (10.3 Hz) placed C12 in an equatorial position and the hemiacetal hydroxyl group was confirmed to be axial by NOE correlations to H13 and H15b. The small H12/H13 coupling (2.2 Hz) established a gauche relationship between these protons however J-based configurational analysis was unable to determine fully the configuration at C12 as the H13-C11 and H13-C36 coupling constants could not be determined. An NOE between H10 and H13 however served to put H13 and C11 gauche and confirm the *R* configuration at C12 in agreement with natural salinomycin (Figures S5, S6)**.**

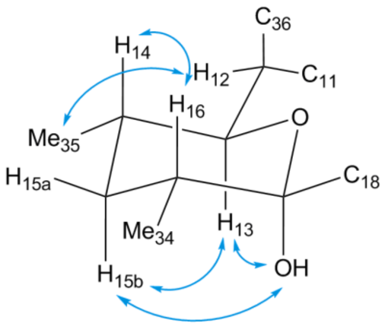

Coupling	$^3J_{H-H}$ /Hz
H12-H13	2.2
H13-H14	10.3
H14-H15a	3.9
H14-H15b	11.6
H15a-H16	3.9
H15b-H16	11.6

Figure S5. Conformation of the B ring (NOE correlations in blue).

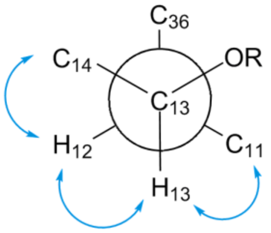

Coupling	J (Hz)	Large / Small
H12-H13	2.2	Small
H12-C13	3.0	Small
H12-C14	3.0	Small
H13-C11	nd	*Small*
H13-C36	nd	*Large*

Expected value for undetermined (nd) coupling constant is shown in *italics*.

Figure S6. Conformation about the C12-C13 bond (NOE correlations in blue). Data fits an A2 or B2 conformer, NOE correlations favour B2.

The remote stereocentre at C19 was linked to that at C17 *via* the diastereotopic protons at C18. First the conformation about the C18/C19 bond was established by J-based configurational analysis. A large (10.6 Hz) coupling placed H18b and H19 in an *anti* orientation and series of small ^3J coupling constants between H18b/C20, H18a/C20 and H18a/H19 indicated that all these pairs were gauche (Figure S7). The small ^2J coupling between H18a/C19 and large ^2J coupling between H18b/C19 confirmed that H18a lay *anti* to the C19 hydroxyl group and thus established the relative configuration of the C18 protons with respect to the C19 stereocentre. The expected NOE correlations for this arrangement were observed (Figure S8). The conformation of the C17/C18 bond was then established. The G-BIRD-HSQMBC spectrum allowed the C16/H18a and C16/H18b coupling constants to be measured and the small values for these indicated gauche relationships in each case (Figure S8). The NOESY spectrum recorded at 700 MHz allowed the identification of NOE correlations between H16/H18b and Me34/H18a which indicated that H18a should be *pro-S* and H18b *pro-R*. Combining the conformation about the C17/C18 bond with that deduced for the

C18/C19 bond, as shown in Figure S8, suggested that H19 should lie close to OH17 and an NOE correlation between these two protons was duly observed. The proposed conformation for this region is shown in Figure S8 and shows that an *S* configuration at C17 can be connected to an *S* configuration at C19 in agreement with the assignment of this centre in the E-15 and E-16 compounds from *S. albus* Δ*salE* mutant (Figure S15) and with the prediction from bioinformatics analysis.[4]

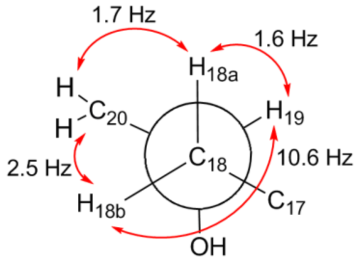

Coupling	J (Hz)	Large / Small
H18a-H19	1.6	Small
H18a-C19	1.5	Small
H18a-C20	1.7	Small
H18b-H19	10.6	Large
H18b-C19	6.5	Large
H18b-C20	2.5	Small
H19-C17	3.1	Small

Figure S7. Conformation about the C18-C19 bond, data fits a D1 conformation.

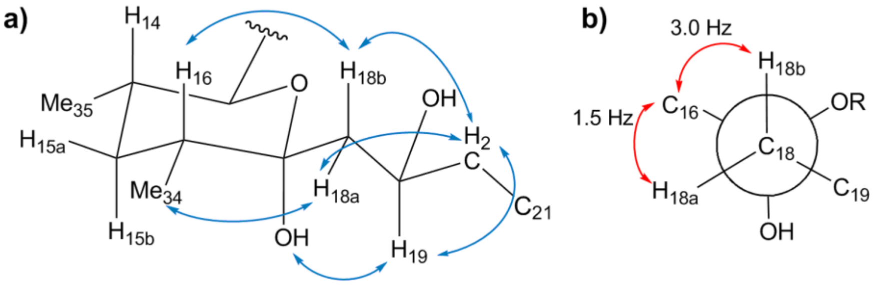

Figure S8. Conformation of the C16-C19 region. **a)** NOE correlations *(note: Flexibility about the C19-C20 bond means that H18a, H18b and H19 each show correlations to both C20 protons)*. **b)** ^{1}H-^{13}C coupling constants between H18 and C16.

The final proposed 3D structure of **3** is shown in Figure S9.

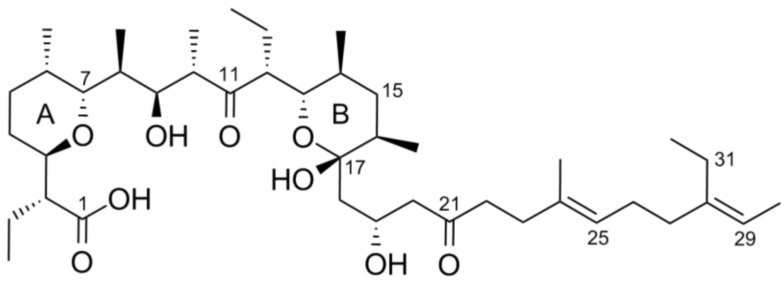

Figure S9. Structure of **3**.

3. Supplementary Tables

Table S1. NMR data for **3**. All spectra were recorded in CD_3CN.

atom	δ ^{13}C, ppm	mult[a]	δ ^{1}H, ppm	mult[b]	J, Hz	spin system	COSY	HMBC	NOESY
1	179.86	s						2, 3, 41a, 41b	
2	50.54	d	2.96	td	10.8, 3.9	1	3, 41a, 41b	3, 4a, 4b, 41, 42	3, 4b, 5a, 7, 41, 42
3	75.50	d	3.91	dd	10.8, 5.1	1	2, 4a, 4b	2, 4a, 4b, 5a, 5b, 7, 41	2, 4a, 4b, 9(w), 41
4a	20.79	t	1.93	m		1	3, 4b, 5a, 5b	2, 3, 5a, 5b	3, 4b, 5b, 40
4b			1.41	brd	13.6	1	3, 4a, 5a, 5b		2, 3, 4a
5a	26.67	t	1.88	tt	13.9, 3.9	1	4a, 4b, 5b, 6	3, 4a, 4b, 6, 7, 40	2, 5b, 6, 7
5b			1.50	m		1	4a, 4b, 5a, 6		4a, 5a, 6, 40
6	28.69	d	1.18	qq	7.0, 3.5	1	5a, 5b, 7, 40	4a, 4b, 5a, 5b	5a, 5b, 7, 39, 40
7	72.87	d	3.53	dd	10.1, 2.4	1	6, 8	3, 8, 9, 39, 40	2, 5a, 6, 8, 9, 39, OH9
8	36.91	d	1.48	dq	10.1, 6.9	1	7, 9 (no tocsy), 39	6, 7, 9, 39	7, 9, 38, 39
9	70.70	d	4.32	ddd	9.9, 4.2, 1.2	2	8 (no tocsy), 10, OH9	7, 10, 38, 39	3, 7, 8, 10, 38, 39
10	48.68	d	2.89	dq	10.2, 7.2	2	12, 38	9, OH9, 38	9, 12, 13, 38, 39
11	216.36	s						9, 10, 12, 13, 36a, 36b, 37	
12	55.21	d	2.78	dt	11.5, 2.3	3	13, 36a, 36b	13, 36a, 36b, 37	10, 13, 35, 36a, 36b, 37
13	74.10	d	4.04	dd	10.3, 2.1	3	12, 14	12, 14, 15b, 18a, 18b, 35	10, 12, 14, 15b, 35 OH17
14	32.88	d	1.68	m		3	10, 15a, 15b, 35	13, 15a, 15b, 34, 35	13, 15a, 16, 35
15a	37.78	t	1.51	dt	12.2, 3.9	3	14, 15b, 16	13, 14, 16, 34, 35	14, 15b, 34, 35
15b			1.31	q	11.6	3	14, 15a, 16		13, 15a, 16, 34, 35, OH17
16	39.66	d	1.54	m		3	15a, 15b, 34	14, 15a, 15b, 18a, 18b, OH17	14, 16, 18b, 34
17	100.59	s						13, 15a, 15b, 16, 18a, 18b, 19, 34, OH17	
18a	44.51	t	1.92	dd	14.5, 1.8	4	18b, 19	16, 19, 20a, 20b	18b, 19, 20a, 20b, 34
18b			1.47	dd	14.5, 10.7	4	18a, 19		16, 18a, 19(w), 20a, 20b
19	64.84	d	4.48	dtd	10.7, 6.4, 1.8	4	18a, 18b, 20a, 20b	18a, 18b, 20a, 20b	18a, 18b(w), 20a, 20b, 22, OH9, OH17
20a	50.22	t	2.64	dd	15.5, 6.6	4	19, 20b	18a, 18b, 19, 22	18a(w), 18b, 19, 20b, 22, 23
20b			2.43	dd	15.5, 6.2	4	19, 20a		18a, 18b, 19, 20a, 22
21	210.73	s						19, 20a, 20b, 22, 24	
22	43.38	t	2.52	t	7.6	5	23	23	19, 20a, 20b, 23, 25, 33
23	33.82	t	2.18	t	7.6	5	22	22, 25, 33	20a, 22, 25, 33
24	134.77	s						22, 23, 26, 33	
25	125.55	d	5.11	tq	6.8, 1.3	6	26, 33	23, 26, 27, 33	22, 23, 26, 27
26[c]	27.48	t	2.04	q	7.5	6	25, 27	25, 27, 33	25, 27, 29, 30, 32[c]
27	37.22	t	1.98	tq	7.2, 1.1	6	26, 29, 31	25, 26, 29, 31	25, 26, 29, 31
28	142.52	s						26, 27, 30, 31, 32	
29	118.77	d	5.17	q	6.7	6	26, 30	27, 30, 31	26, 27, 30, 31
30	13.14	q	1.56	brd	6.6	6	29	29, 31	26, 29, 31
31[c]	23.29	t	2.04	q	7.5	7	26, 32	27, 29, 30	25, 27, 29, 30, 32[c]
32	13.12	q	0.94	t	7.6	7	31	29, 31	26/31
33	16.12	q	1.57	brs		6	25	23, 25	22, 23
34	16.63	q	0.82	d	6.4	3	16	15a, 15b, 16	15a, 15b, 16, 18a, OH17
35	17.21	q	0.87	d	6.7	3	14	13, 14, 15a, 15b	12, 13, 14, 15a, 15b
36a	16.08	t	1.7	tq	11.5, 7.4	3	12, 36b, 37	12, 13, 17	12, 36b, 37
36b			1.35	dqd	11.5, 7.4, 2.3	3	12, 36a, 37		12, 36a, 37
37	12.67	q	0.76	t	7.4	3	36a, 36b	12, 36a, 36b	12, 36a, 36b
38	13.47	q	0.76	d	7.2	2	10	9, 10	8, 9, 10
39	7.36	q	0.72	d	6.9	1	8	7, 8, 9	6, 7, 8, 9, 40, OH9
40	11.23	q	0.93	t	7	1	6	5a, 5b, 6, 7	4a, 5b, 6, 39
41a	23.65	t	1.5	m		1	2, 41b, 42	2, 3, 42	2, 3, 42
41b			1.46	m		1	2, 41a, 42		2, 3, 42
42	12.31	q	0.69	t	7.3	1	41a, 41b	2, 41	2, 41
OH 1			11.50	brs					
OH 9			3.27	brs		2	9		7, 19, 39
OH17			3.68	brs					13, 15b, 19, 34(w)
OH19			4.31	brs					

[a] Determined from DEPT and multiplicity (mult) edited HSQC spectra, s – singlet, d – doublet, t – triplet, q – quartet. [b] s – singlet, d – doublet, t – triplet, q – quartet, br – broad, m – multiplet, w – a weak coupling. [c] The signals for H26 and H31 overlapped and couplings to these in COSY and NOESY spectra could not be definitively assigned.

Table S2. List and description of vectors used. Antibiotic resistance markers are highlighted in bold.

Name	Description	Application	Reference
pYH7	***tsr***, ***bla***, ***aac(3)IV***, *cos*, *oriT*, P_{T7}, P_{T3}, ori_{pIJ101}, ori_{ColEI}	in-frame gene deletion in *Streptomyces* by homologous recombination	[2]
pIB139	***aac(3)IV***, *oriT*, *attP* (ΦC31), *int*, P_{ermE*}, ori_{pUC}	integration/complementation of mutant strains *in trans*	[5]
pET-29b(+)	***neo***, P_{T7}, ori_{pBR322}, *lacI*, C-terminal His_6-tag, ori_{F1}	overexpression of genes in *E. coli*	Novagen
pHLΔ*salBIII*	pYH7-derived, contains regions of homology to the right and to the left of *salBIII* gene	construct for *salBIII* gene in-frame deletion	this study
pIB-*salBIII*	pIB139-derived, *salBIII* gene cloned	complementation of Δ*salBIII*	this study
pET-SalBIII_C	pET-29b(+)-derived, *salBIII* gene cloned with C-terminal His-tag (*Nde*I/*EcoR*I)	overexpression of *salBIII*	this study
pIB*salBIII*_Tyr14Ala	derived from pIB-*salBIII*, SalBIII has Tyr14Ala mutation	complementation of Δ*salBIII* mutant	this study
pIB*salBIII*_Tyr54Ala	derived from pIB-*salBIII*, SalBIII has Tyr54Ala mutation	complementation of Δ*salBIII* mutant	this study
pIB*salBIII*_Asn58Ala	derived from pIB-*salBIII*, SalBIII has Asn58Ala mutation	complementation of Δ*salBIII* mutant	this study
pIB*salBIII*_Asn58Asp	derived from pIB-*salBIII*, SalBIII has Asn58Asp mutation	complementation of Δ*salBIII* mutant	this study
pIB*salBIII*_Asp38Ala	derived from pIB-*salBIII*, SalBIII has Asp38Ala mutation	complementation of Δ*salBIII* mutant	this study
pIB*salBIII*_Asp38Asn	derived from pIB-*salBIII*, SalBIII has Asp38Asn mutation	complementation of Δ*salBIII* mutant	this study
pIB*salBIII*_Asp104Ala	derived from pIB-*salBIII*, SalBIII has Asp104Ala mutation	complementation of Δ*salBIII* mutant	this study
pIB*salBIII*_Asp104Asn	derived from pIB-*salBIII*, SalBIII has Asp104Asn mutation	complementation of Δ*salBIII* mutant	this study
pIB*salBIII*_Ile65Ala	derived from pIB-*salBIII*, SalBIII has Ile65Ala mutation	complementation of Δ*salBIII* mutant	this study
pIB*salBIII*_Arg45Ala	derived from pIB-*salBIII*, SalBIII has Arg45Ala mutation	complementation of Δ*salBIII* mutant	this study
pIB*salBIII*_Trp121Ala	derived from pIB-*salBIII*, SalBIII has Trp121Ala mutation	complementation of Δ*salBIII* mutant	this study

Table S3. List and description of the strains used.

Strain	Genotype/Description	Reference
***E. coli* strains**		
DH10B	F^- *endA1 recA1 galE15 galK16 nupG rpsL* Δ*lacX74* Φ80*lacZ*ΔM15 *araD139* Δ(*ara*,*leu*)7697 *mcrA* Δ(*mrr-hsdRMS-mcrBC*) λ^- ; Host for general DNA manipulations	Invitrogen
BL21(DE3)	F^- *ompT gal dcm lon hsd*S_B(r_B^- m_B^-) λ(DE3 [*lacI lacUV5-T7 gene 1 ind1 sam7 nin5*]); Host for gene expression	Novagen
ET12567 (pUZ8002)	(F^- *dam*-13::Tn9 *dcm*-6 *hsd*M *hsd*R recF143 *zjj*-202::Tn10 *gal*K2 *gal*T22 *ara*14 *pac*Y1 *xyl*-5 *leu*B6 *thi*-1); pUZ8002: *tra*, *neo*, RP4 Donor strain for conjugation between *E. coli* and *Streptomyces*	[19]
***Streptomyces* strains**		
S. albus		
DSM 41398	wild type strain producing salinomycin	DSMZ
DSM 41398 Δ*salC*	*salC* gene in-frame deletion mutant	[6]
DSM 41398 Δ*salBIII*	*salBIII* gene in-frame deletion mutant	this study
DSM 41398 Δ*salC*/Δ*salBIII*	*salC and salBIII* genes double mutant	this study

Table S4. Oligonucleotide primers used and corresponding annealing temperatures (T_{an}). Restriction and mutated sites are underlined or in bold, respectively.

**Name**	**Sequence, 5' – 3' direction**	**T_{an}, °C**
Primers used for construction of in-frame gene deletion plasmid		
SalBIII_1F	TGATCAAGGCGAATACTTCATATGCGAGGAGAAGGTCGGCGTCTTCGC	72 (-0.2)*
SalBIII_1R	CGATCTTGCCCTCATCGCCCTCGTTCACCTTGCGGA	72 (-0.2)*
SalBIII_2F	ACGAGGGCGATGAGGGCAAGATCGACAGCATGCGCG	72 (-0.2)*
SalBIII_2R	CCGCGCGGTCGATCCCCGCATATGGCAGCGCCGGTATCGCGTTCATGA	72 (-0.2)*
Primers used for PCR confirmation of gene deletion		
PCR salBIII_f	CTGACAGCACGTCAGCCGGTGTC	72 (-0.2)*
PCR salBIII_r	GTAGAGCGCGATGAGGAGGTTCTC	72 (-0.2)*
Primers used for cloning of salBIII gene into pIB139 vector		
salBIII_*Nde*I_F	GACCATATGCAGGACGAGCAGAAGCG	63
salBIII_*Eco*RV_R	GCGGATATCTCAGACGCCGATGTCGGTCA	63
Primers used for site directed mutagenesis in the salBIII gene		
SalBIII_Tyr14-Phe_F	CGCAAGGAGATCGTCGCGGAA**TTC**TTCCGCAAGGTGAACGAG GGC	72
SalBIII_Tyr14-Phe_R	GCCCTCGTTCACCTTGCGGAA**GAA**TTCCGCGACGATCTCCTTGCG	72
SalBIII_Tyr54-Phe_F	GGCCGCGCAGCGCAGCGCGAG**TTC**TTCAACAGCAATGTCACCGCC	72
SalBIII_Tyr54-Phe_R	GGCGGTGACATTGCTGTTGAA**GAA**CTCGCGCTGCGCTGCGCGGCC	72
SalBIII_Asn58-Ala_F	CAGCGCGAGTACTTCAACAGC**GCT**GTCACCGCCGAGGTCACCATC	72
SalBIII_Asn58-Ala_R	GATGGTGACCTCGGCGGTGAC**AGC**GCTGTTGAAGTACTCGCGCTG	72
SalBIII_Asn58-Asp_F	CAGCGCGAGTACTTCAACAGC**GAT**GTCACCGCCGAGGTCACCATC	72
SalBIII_Asn58-Asp_R	GATGGTGACCTCGGCGGTGAC**ATC**GCTGTTGAAGTACTCGCGCTG	72
SalBIII_Asp38-Ala_F	ACCGAGAACGCCACCATCGAG**GCC**CCGGTCGGCAAGGATGTCCGC	72
SalBIII_Asp38-Ala_R	GCGGACATCCTTGCCGACCGG**GGC**CTCGATGGTGGCGTTCTCGGT	72
SalBIII_Asp38-Asn_F	ACCGAGAACGCCACCATCGAG**AAC**CCGGTCGGCAAGGATGTCCGC	72
SalBIII_Asp38-Asn_R	GCGGACATCCTTGCCGACCGG**GTT**CTCGATGGTGGCGTTCTCGGT	72
SalBIII_Asp104-Ala_F	CGTGTGAAGATCAACGCCGTC**GCC**GTCTTCACCCTCACCCCCGAG	72
SalBIII_Asp104-Ala_R	CTCGGGGGTGAGGGTGAAGAC**GGC**GACGGCGTTGATCTTCACACG	72
SalBIII_Asp104-Asn_F	CGTGTGAAGATCAACGCCGTC**AAC**GTCTTCACCCTCACCCCCGAG	72
SalBIII_Asp104-Asn_R	CTCGGGGGTGAGGGTGAAGAC**GTT**GACGGCGTTGATCTTCACACG	72
SalBIII_Ile65-Thr_F	AATGTCACCGCCGAGGTCACC**ACC**GAGCCGGGACACCTGTCGGCG	72
SalBIII_Ile65-Thr_R	CGCCGACAGGTGTCCCGGCTC**GGT**GGTGACCTCGGCGGTGACATT	72
SalBIII_Arg45-Ala_F	GACCCGGTCGGCAAGGATGTC**GCC**GAGGGCCGCGCAGCGCAGCGC	72
SalBIII_Arg45-Ala_R	GCGCTGCGCTGCGCGGCCCTC**GGC**GACATCCTTGCCGACCGGGTC	72
SalBIII_Trp121-Ala_F	ATCGACAGCATGCGCGTGTTC**GCG**GGCATGACCGACATCGGCGTC	72
SalBIII_Trp121-Ala_R	GACGCCGATGTCGGTCATGCC**CGC**GAACACGCGCATGCTGTCGAT	72
Primers used for cloning of salBIII gene into pET29b(+) vector		
salBIII_*Nde*I_F	GACCATATGCAGGACGAGCAGAAGCG	63
salBIII_*Eco*RI_R	GCGGAATTCCAGACGCCGATGTCGGTCAT	63

* Each subsequent cycle annealing temperature was lowered by 0.2°C.

Table S5. HRMS data for **3** ([M+Na]+ and [M+NH4]+ ions).

Formula	calc. m/z	det. m/z	Error [ppm]
$C_{42}H_{72}O_9Na^+$	743.5069	743.5069	0
$C_{42}H_{76}NO_9^+$	738.5515	738.5522	0.95

Table S6. HRMS and HRMS/MS data for compound **2** ([M+Na]+ ion).

Formula	calc. m/z	det. m/z	Error [ppm]
$C_{42}H_{74}O_{10}Na^+$	761.5174	761.5170	-0.53
$C_{42}H_{72}O_9Na^+$	743.5069	743.5065	-0.54
$C_{42}H_{70}O_8Na^+$	725.4963	725.4959	-0.55
$C_{38}H_{66}O_8Na^+$	673.4650	673.4648	-0.30
$C_{28}H_{50}O_8Na^+$	553.3347	553.3344	-0.54
$C_{28}H_{48}O_7Na^+$	535.3241	535.3241	0
$C_{27}H_{50}O_8Na^+$	525.3398	525.3396	-0.38
$C_{27}H_{48}O_7Na^+$	507.3292	507.3290	-0.39
$C_{29}H_{50}O_5Na^+$	501.3550	501.3549	-0.20
$C_{29}H_{48}O_4Na^+$	483.3445	483.3442	-0.62
$C_{23}H_{38}O_4Na^+$	401.2662	401.2659	-0.75
$C_{19}H_{36}O_6Na^+$	383.2404	383.2401	-0.78
$C_{19}H_{34}O_5Na^+$	365.2298	365.2297	-0.27
$C_{13}H_{24}O_5Na^+$	283.1516	283.1512	-1.41

Table S7. HRMS^3 data for compound **2** ([M+Na]+ ion, 761.5→383.2).

Formula	calc. m/z	det. m/z	Error [ppm]
$C_{19}H_{36}O_6Na^+$	383.2404	383.2400	-1.04
$C_{19}H_{34}O_5Na^+$	365.2298	365.2295	-0.82
$C_{19}H_{32}O_4Na^+$	347.2193	347.2187	-1.73
$C_{13}H_{24}O_5Na^+$	283.1516	283.1512	-1.41
$C_{13}H_{22}O_4Na^+$	265.1410	265.1408	-0.75

Table S8. HRMS and HRMS/MS data for compound **B-11** ([M+Na]+ ion).

Formula	calc. m/z	det. m/z	Error [ppm]
$C_{42}H_{72}O_{12}Na^+$	791.4916	791.4905	-1.39
$C_{42}H_{70}O_8Na^+$	773.4810	773.4801	-1.16
$C_{38}H_{64}O_{10}Na^+$	703.4392	703.4384	-1.14
$C_{29}H_{50}O_8Na^+$	549.3398	549.3392	-1.09
$C_{29}H_{48}O_7Na^+$	531.3292	531.3286	-1.13
$C_{29}H_{46}O_6Na^+$	513.3187	513.3181	-1.17
$C_{23}H_{36}O_6Na^+$	431.2404	431.2397	-1.62
$C_{23}H_{34}O_5Na^+$	413.2298	413.2292	-1.45
$C_{19}H_{36}O_6Na^+$	383.2404	383.2396	-2.09
$C_{13}H_{24}O_5Na^+$	283.1516	283.1509	-2.47
$C_{13}H_{22}O_4Na^+$	265.1410	265.1403	-2.64

Table S9. Restoration of salinomycin (**1**) production in *S. albus* Δ*salBIII* by wild type (WT) and mutant forms of SalBIII.

**SalBIII variant**	**Relative level of restoration of compound 1 production**
WT	+++
Tyr14Phe	++
Asp38Ala	–
Asp38Asn	–
Tyr54Phe	++
Asn58Ala	++
Asn58Asp	–
Ile65Thr	++
Asp104Ala	–
Asp104Asn	–
Trp121Ala	+
Arg45Ala	+++

Table S10. Statistics of data collection, refinement and validation of SalBIII.

PDB ID	5CXO
X-ray diffraction data and refinement	
Beamline	PETRA III, P13
Wavelength (Å)	0.977237
Resolution (Å)	48.57-1.8
Space group	$I2_12_12_1$
Unit cell (Å)	
a, b, c	53.0 101.9 120.8
α, β, γ (°)	90.0, 90.0, 90.0
Resolution range (Å)	48.57-1.8
Highest resolution shell (Å)	1.9-1.8
R_{sym}[a](%) (*)	3.7 (36.2)
Completeness (%) (*)	98.9 (98.7)
Number of unique reflections (*)	30391 (4367)
Multiplicity (*)	13.1 (13.5)
Average intensity, $\langle I/\sigma(I)\rangle$ (*)	37.0 (6.8)
Subunits in the asymmetric unit (A.U.)	2
R.M.S.D. of the subunits in the A.U.	0.78
R_{cryst}[b]	21.09
R_{free}[c]	27.52
Number of non-hydrogen atoms	2276
Number of Polyethylene glycol (PEG) fragments	5
Number of water molecules	163
B-factor analysis (Å^2)	
Main chain	35.1
Side chain	41.1
PEG fragments	52.9
Water molecules	47.4
Ramachandran plot	
Most favored	90.5
Additional allowed	9.5
Generously allowed	0
Disallowed	0
R.M.S D. of ideal geometry	0.022
Bond (Å)	2.336

(*) the values in parenthesis refer to the highest resolution shell

[a]$R_{sym} = \Sigma_h|I_h - \langle I\rangle|/\Sigma_h I_h$, where $I_h$is the intensity of reflection h $\langle I\rangle$ is the mean intensity of all symmetry-related reflections.

[b]$R_{factor} = \Sigma||F_{obs}| - |F_{cal}||/\Sigma|F_{obs}|$, where F_{calc} and F_{obs} are observed and calculated structure factor amplitudes

[c]$R_{free} = R_{factor}$, but using a random subset of the data (5%) which is excluded from the refinement.

4. Supplementary Figures

```
                    10        20        30        40        50        60
LasB_N    -----------------MPAETVRKEVALEYCRRVNAGELEGVLQLFAPDARLVDPLGTE
LasB_C    -------------------DEERRKELAREHCLRINDGDVDGLLKLYSPRIRFEDPVGSW
NanI_N    -----------------MLNERTRKKIAREHSRRLSEGDIDGLLDLYAKDVTFEAPVGAG
NanI_C    ------------------PDEAALRELPHNYLRLLQKGDVEGTVALFTDDIVFEDPVGGL
MonBI     ------------------MNEFARKKRALEHSRRINAGDLDAIIDLYAPDAVLEDPVGLP
MonBII    -----------------MPDEAARKQMAVDYAERINAGDIEGVLDLFTDDIVFEDPVGRP
NigBI     -----------------MIDERSRKKLILEHSRRMNAADVDGLLELYADEVTFEDPVGSG
NigBII    -----------------MADEATLKRMALEYARRMNTGDVEAVLDLFSDDIVFEDPVGAP
TmnB      -----------------MLDEDARKRVALEYCRRIGAGDVDGVLALFADPLHVEDPVGDK
TsnB      -----------------MPDEAERKKVALEYCHRLNAGDVDGALALFSPEIRYEDPVGSG
LEH       MTSKIEQPRWASKDSAAGAASTPDEKIVLEFMDALTSNDAAKLIEYFAEDTMYQN-MPLP
SalBI     -----------------MLDEATRKELALEHCRRMTSGDVDHMLGLYTKDVRFEDPVGSV
SalBII    -----------------MLDEAALKELILEHTRRVNAGDVEGLLKLYSPEVRFEDPVGAG
SalBIII   -----------------MQDEQKRKEIVAEYFRKVNEGDVDAIVEMFTENATIEDPVGKD

                    70        80        90       100       110       120
LasB_N    PVVGRAALAARLAPALRGAVHEEPGRPYAAHDGTSVVLPATVTVG---------------
LasB_C    TRTGLEALRAHATMAVGSNVRETAGLTVAGQDGRHAAVTVSATMDYLPSGPLLARHHLMT
NanI_N    GQAGHEALRAHFESAVAGNVDETIVESVVGQDGEHVLSRITAVMDYRPRGPLYADRGW--
NanI_C    LLRGKDALREHAFRGSEGKVHEMLGRLVTSMDGRFVVVLGDARVY---------------
MonBI     PVTGHDALRAHYEPLLAAHLREEAAEPVAGQDATHALIQISSVMDYLPVGPLYAERGW--
MonBII    PMVGKDDLRRHLELAVSCGTHEVPDPPMTSMDDRFVVTPTTVTVQ---------------
NigBI     RRTGRDALRAHFEELAAAGIAEIPGEPVAGQDGVHVLVPVTATMDYLPKGPGFAERGW--
NigBII    PLIGKDALREHIAWSIECQVHETPGRPVTSMDGRRVAVPTTVTVY---------------
TmnB      TFTARAEYRAHIARLVSYGVHEVPGTPVAGLDREHVALPITVELR---------------
TsnB      TLTGLTALREHLARAVASRVLETPGVPVASLDDEHVLLPATAELD---------------
LEH       PAYGRDAVEQTLAGLFTVMSIDAVETFHIGSSNGLVYTERVDVLR---------------
SalBI     PMIGHDELRPHFQRAAEAKTVDVAGIPVAAQDGIHVAIPVAVYMNYLPLGPTLTKHGY--
SalBII    AQQGHEALRAHATGAVTSNAVEIAGRPVAAQDGLHAAVPVTATMDYLPLGPVLTRYGV--
SalBIII   VREGRAAQREYFNSNVTAEVTIEPGHLSAGQDGKSVAVALAAEMT---------------

                   130       140       150       160       170       180
LasB_N    --APGAPPQRRGRTRVMGVIEVGEDGLIREMRVMWGVTDSSWTARPAPDEERRKE-----
LasB_C    LPAPADPHRALIGIEYVMVIGVDADGLIDEMRAYWGATDVSLLDPAA-------------
NanI_N    LPAPEGAEPTALRCHYALLLRVGESGLIEDMRAYWGKPDLE-------------------
NanI_C    --VP-----ARMRMRMITICEVNEDRLGAHIQGFWGLTDMTIGFPDDDTAPEPALDTARR
MonBI     LKAPDAPGTARIHRTAMLVIRMDASGLIRHLKSYWGTSDLTVLG----------------
MonBII    --RP-----RPMTFRIVGIVELDEHGLGRRVQAFWGVTDVTMDDPAGPADTTHPEGIRA-
NigBI     LAAPDTPESSRITWDYVLMMRAGAGGLIQELQMFWGRSDIAIAG----------------
NigBII    --AP-----AKLTFSIIGVIELGDDGLVHHAQAFWGITDTKVGDGPELTGVAHFMAVTQN
TmnB      --PEGVPQGKIVRISLIAVMRIGGDGLIRHMRVISGRTDMSLHDAAEAADAARFM-----
TsnB      --DARLPAGSRLTVAFIALLRVGADGLIREMRLFWGRTDTSVTPAASAAQGVR-------
LEH       ----ALPTGKSYNLSILGVFQLT-EGKITGWRDYFDLREFEEAVDLPLRG----------
SalBI     LDKPTNPFQKRIKFDLTSIFRTGSHGLIEAVWVYWGKSDLSLLDPGEE------------
SalBII    LPTPADPTGQRMQFQFLMVIRAGADGLIEDMRAYWGTSDVALVDAAQGGAHA--------
SalBIII   --NILDPNRTRVKINAVDVFTLTPEGKIDSMRVFWGMTDIGV------------------

                   190
LasB_N    --------------
LasB_C    --------------
NanI_N    --------------
NanI_C    PERKDALKPFGTPS
MonBI     --------------
MonBII    --------------
NigBI     --------------
NigBII    LAKMVQAKGSPSPM
TmnB      --------------
TsnB      --------------
LEH       --------------
SalBI     --------------
SalBII    --------------
SalBIII   --------------
```

Figure S10. Multiple sequence alignment of the polyether epoxide hydrolases, limonene epoxide hydrolase (LEH) and SalBIII. The catalytic amino acid dyad is marked by red star and highlighted in yellow. The following proteins were used for alignment: Lasalocid LasB[20] (contains two domains LasB-N and LasB-C), nanchangmycin NanI[21] (contains two domains NanI-N and NanI-C), monensin MonBI and MonBII,[22] nigericin NigBI and NigBII,[23] tetronomycin TmnB,[24] tetronasin TsnB (Dr. F. Huang, personal communication), LEH,[25] salinomycin SalBI and SalBII.[6] Alignment was compiled by the Multalin program.[26]

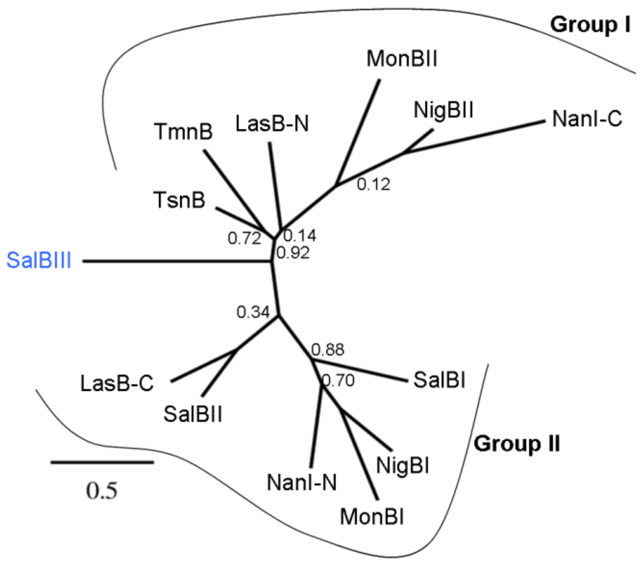

Figure S11. Phylogenetic analysis of polyether epoxide hydrolases. Lasalocid LasB,[20] nanchangmycin NanI,[21] monensin MonBI and MonBII,[22] nigericin NigBI and NigBII,[23] tetronomycin TmnB,[24] tetronasin TsnB (Dr. F. Huang, personal communication), and salinomycin SalBI, SalBII, and SalBIII[6] epoxide hydrolases are shown. PhyML 3.1/3.0 program was used for the phylogenetic tree construction.[27]

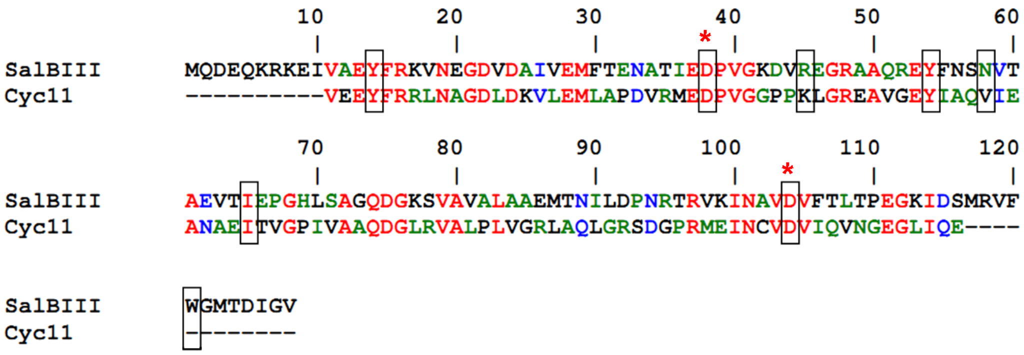

Figure S12. Sequence alignment of SalBIII and Cyc11 domain from indanomycin[28] biosynthesis. Amino acid residues proposed to constitute the active site cavity are shown in boxes. Asp38/28 and Asp104/94 are proposed to be a catalytic dyad (marked by red star). Alignment was compiled by the Multalin program.[26]

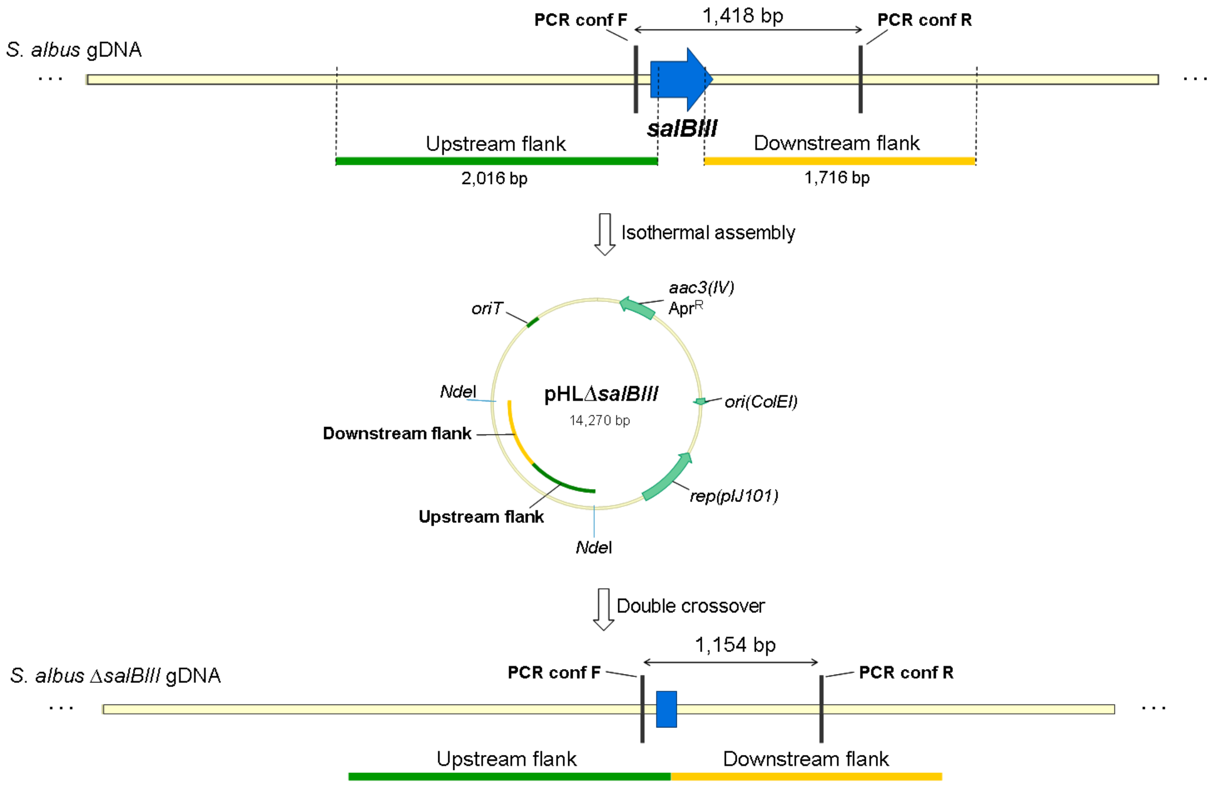

Figure S13. Schematic illustration of the in-frame deletion of the *salBIII* gene in the salinomycin biosynthetic gene cluster. *SalBIII* and its truncated sequence are shown in blue.

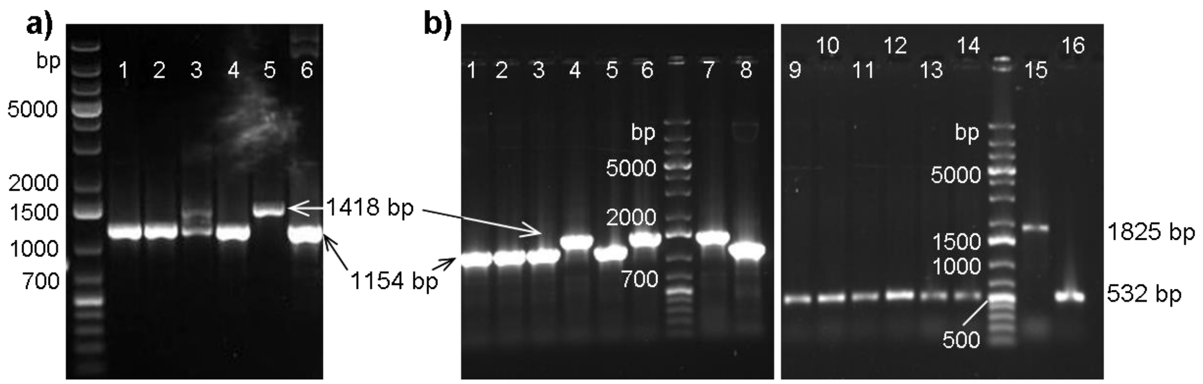

Figure S14. PCR analysis of in-frame deletion of *salBIII* gene in the salinomycin biosynthetic gene cluster in *S. albus* WT **(a)** and *S. albus* Δ*salC* **(b)** strains. For the *salBIII* gene knockout, a band of 1,154 bp is expected, whereas for reversion to WT, a band of 1,418 bp is expected. **a)** Lanes 1, 2, 3 and 4 correspond to the PCRs with gDNA of potential mutant colonies, lane 5 – negative control (WT gDNA was used in the PCR), lane 6 – positive control (plasmid pHLΔ*salBIII* was used as a DNA template in the PCR). **b)** Lanes 1, 2, 3, 4, 5 and 6 correspond to the PCRs with gDNA of potential double mutant colonies with PCR salBIII_f/r primer pair, lane 7 – negative control, lane 8 – positive control. Lanes 9, 10, 11, 12, 13, and 14 correspond to the PCRs with gDNA of of the same potential double mutant colonies, but PCR salC_f/r[6] primer pair was used to confirm that *salC* gene was deleted, lane 7 – negative control, lane 8 – positive control (plasmid pMYΔ*salC*[6] was used as a DNA template in the PCR). If the *salC* gene has been deleted, a band of 532 bp is expected, whereas for the WT strain, a band of 1,825 bp is expected. 1kb Plus DNA Ladder (Fermentas) was used as a molecular DNA size marker.

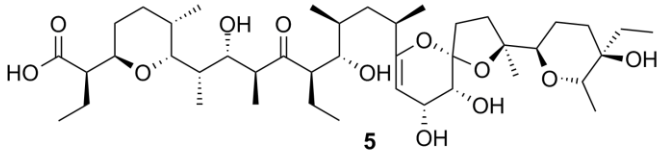

5

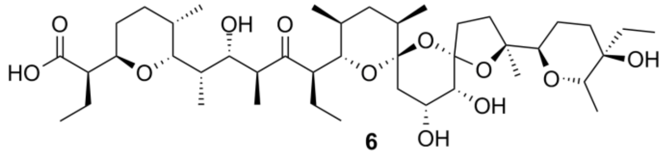

6

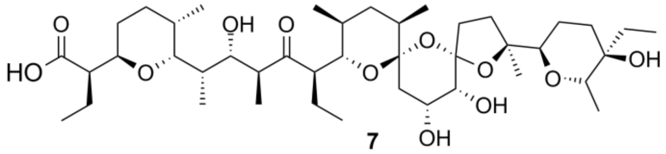

7

Figure S15. Chemical structures of the metabolites from *S. albus* ∆*salE* mutant.[4,29]

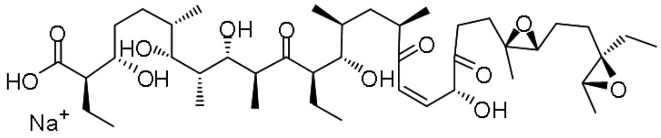

Figure S16. Proposed general structure for the metabolites observed in *S. albus* ∆*salBIII* mutant. The structure is drawn in fully uncyclized form as no conclusion about cyclization pattern and differences between two isomers (**B-8**, **B-11**) can be made from MS^n analysis. Ring A is not present.

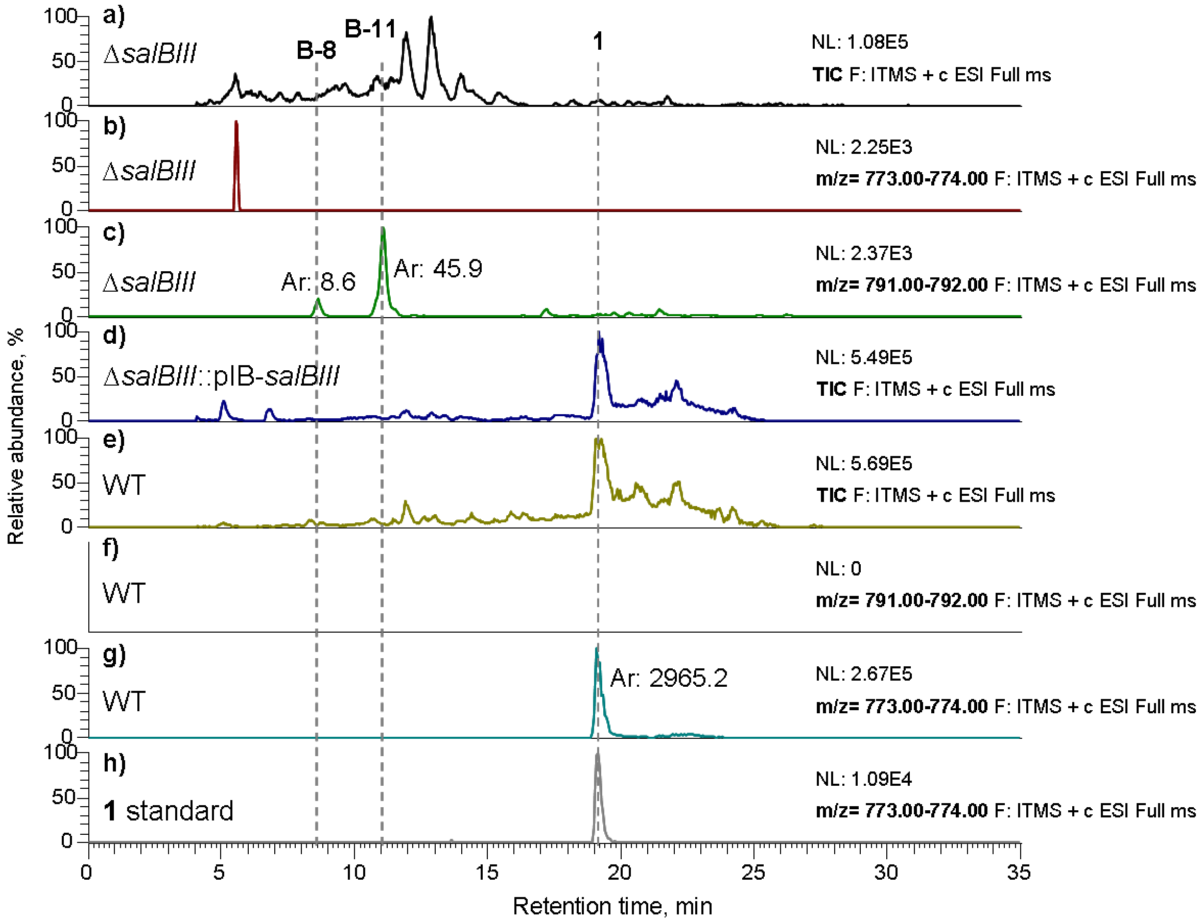

Figure S17. HPLC-MS analysis of the metabolite profile of *S. albus* Δ*salBIII* mutant. **a)** HPLC-MS profile of ethyl acetate extract from *S. albus* Δ*salBIII* mutant, total ion current (TIC). Salinomycin (**1**) production is abolished **(b)**, two novel metabolites at retention time 8.6 and 11.1 min with m/z 791.5 are observed **(c)**. **1** production was fully restored in the *S. albus* Δ*salBIII* mutant complemented with *salBIII* **(d)**. **e)** *S. albus* WT, TIC; no metabolites with m/z 791.5 are detected in the WT extract (**f**). **g)** *S. albus* WT, m/z 773.5. The peak at 20.43 min corresponds to **1** $[M+Na]^+$. **h) 1** standard, m/z 773.5. Relative area (Ar) of the peaks corresponding to **B-8**, **B-11** and **1** is indicated, which shows that production levels of novel metabolites are reduced significantly (around 1.5% the WT production level) as judged by the LC-MS results.

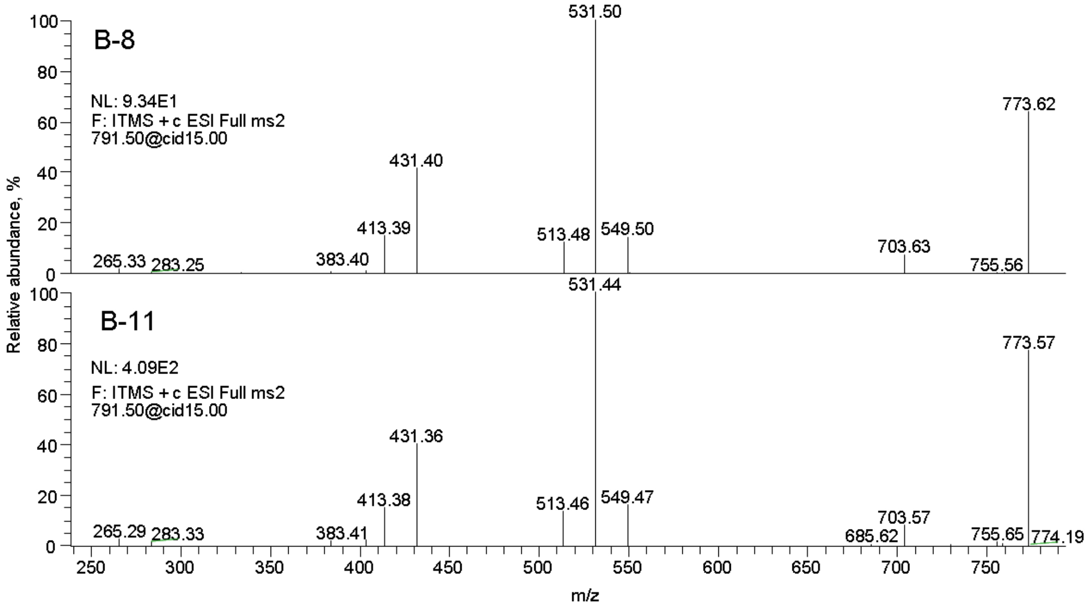

Figure S18. MS/MS spectra of the molecular ion with m/z 791.5 at 8.6 and 11.1 min from *S. albus* Δ*salBIII* mutant metabolite profile. Both compounds show identical MS/MS fragmentation pattern, which suggests that they are structural isomers.

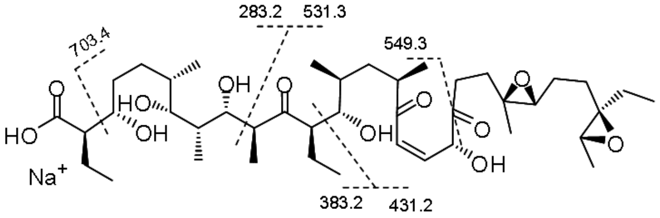

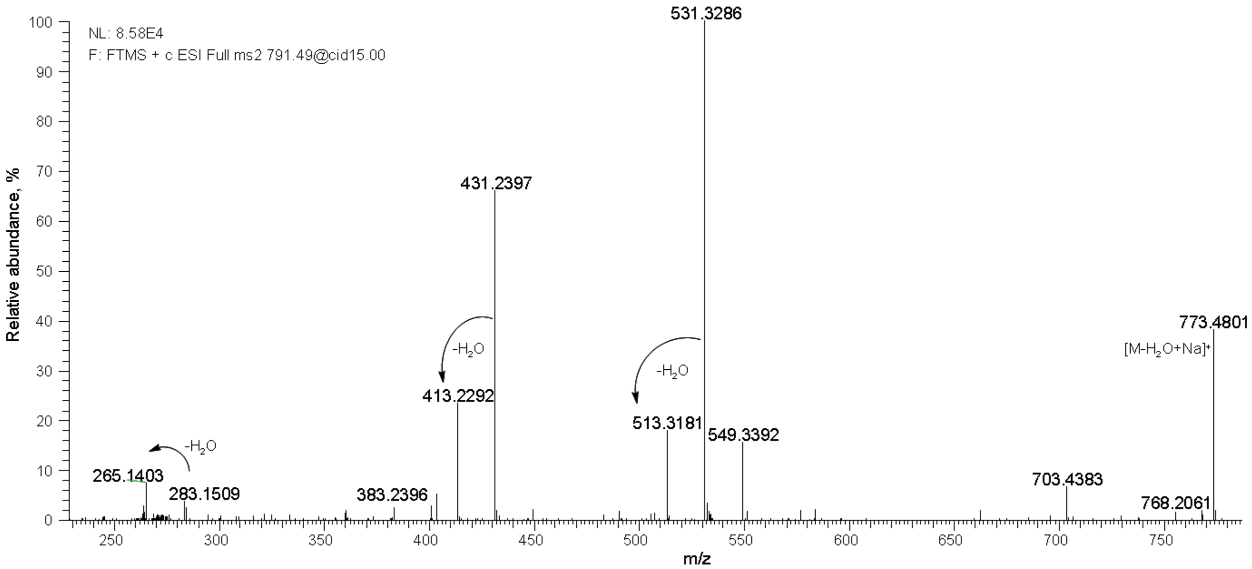

Figure S19. Proposed MS/MS fragmentation pattern and HRMS/MS spectrum of m/z 791.5, compound **B-11** produced by *S. albus* Δ*salBIII*.

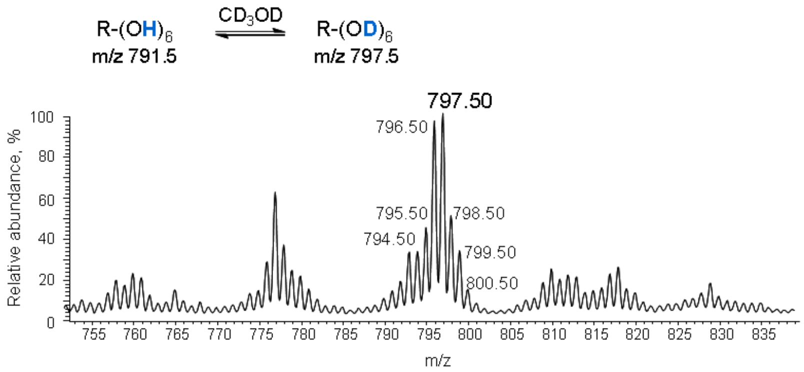

Figure S20. MS spectrum of deuterated [M+Na]$^+$ ions for compound B-11. 6 Da shift in the mass of molecular [M+Na]$^+$ ion at m/z 791.5 suggests the presence of six exchangeable protons in **B-11**.

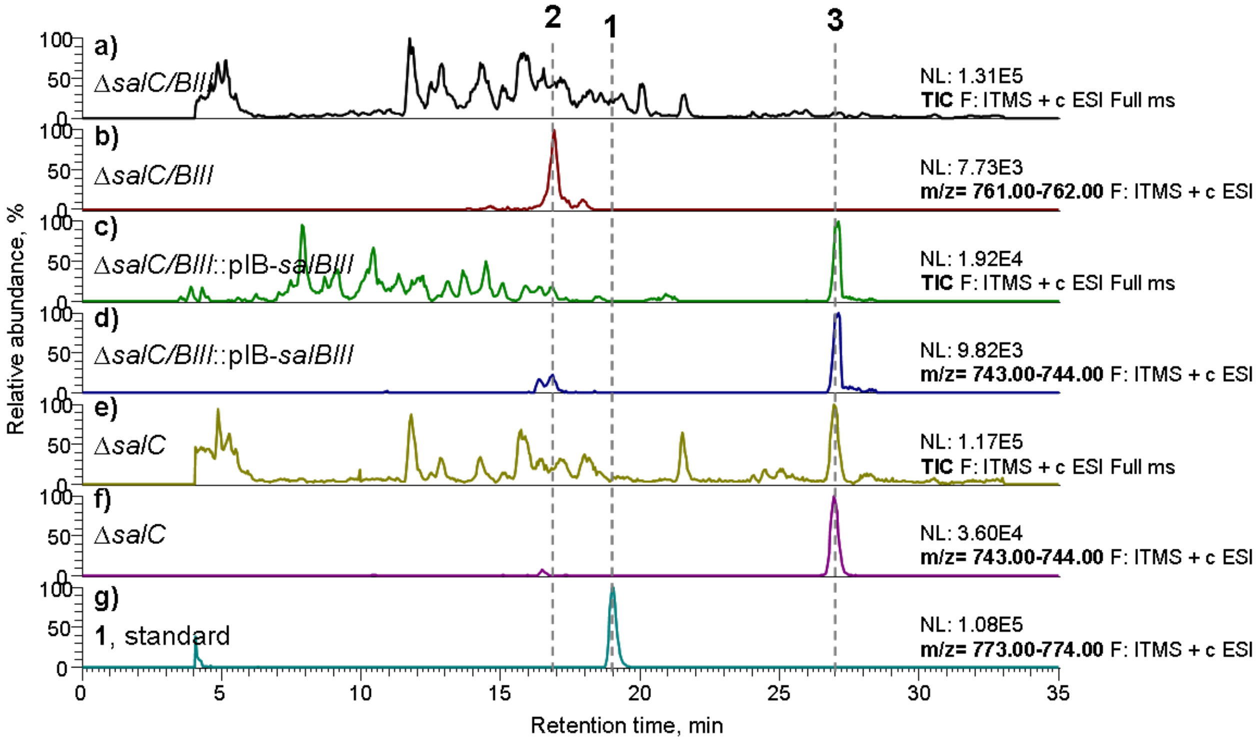

Figure S21. HPLC-MS analysis of the metabolite profile of *S. albus* Δ*salC*/Δ*salBIII* and *S. albus* Δ*salC* mutants. HPLC-MS profile of ethyl acetate extract from *S. albus* Δ*salC*/Δ*salBIII* mutant, total ion current **(a)** and m/z 761.5 **(b)**. **c,d) 3** production was fully restored in the *S. albus* Δ*salC*/Δ*salBIII* mutant complemented with *salBIII*. **e)** *S. albus* Δ*salC* TIC, **f)** *S. albus* Δ*salC* m/z 743.5. **g) 1** standard, m/z 773.5.

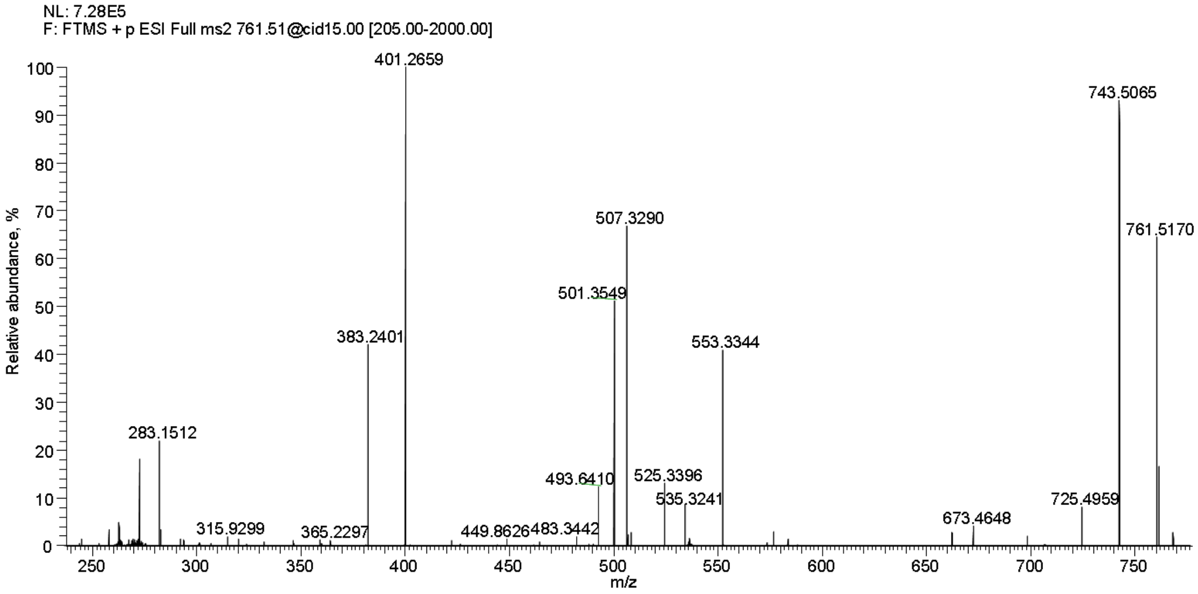

Figure S22. HRMS/MS spectrum of m/z 761.5, compound **2** produced by *S. albus* Δ*salC*/Δ*salBIII*.

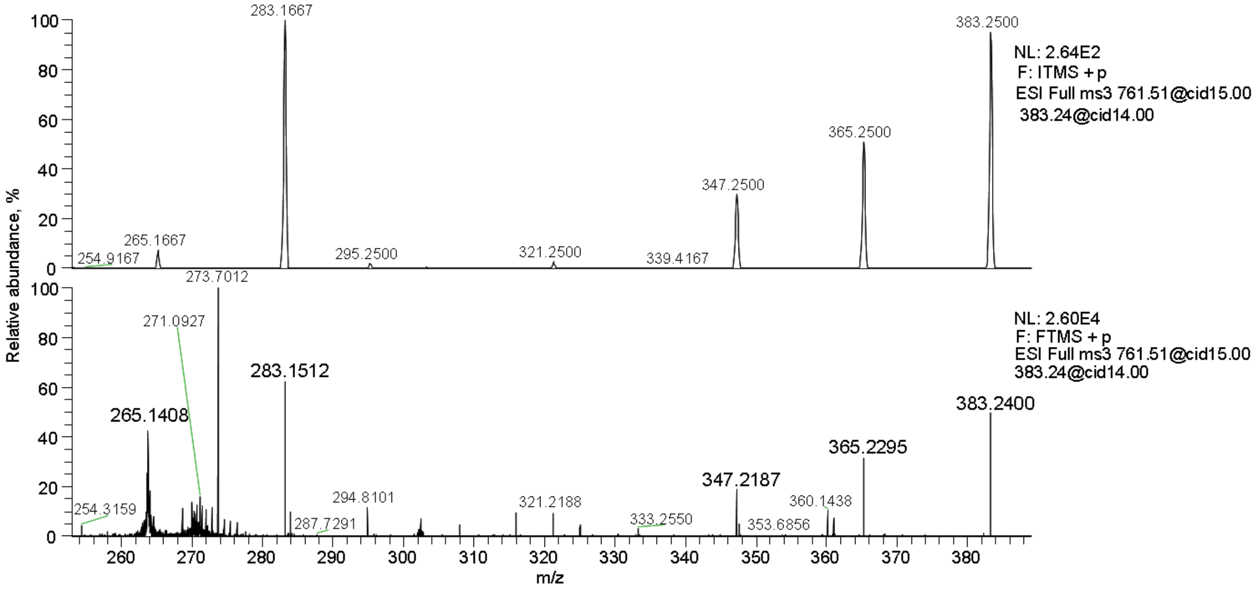

Figure S23. HRMS^3 spectrum of m/z 761.5→383.2 from compound **2** produced by *S. albus* Δ*salC*/Δ*salBIII*.

.

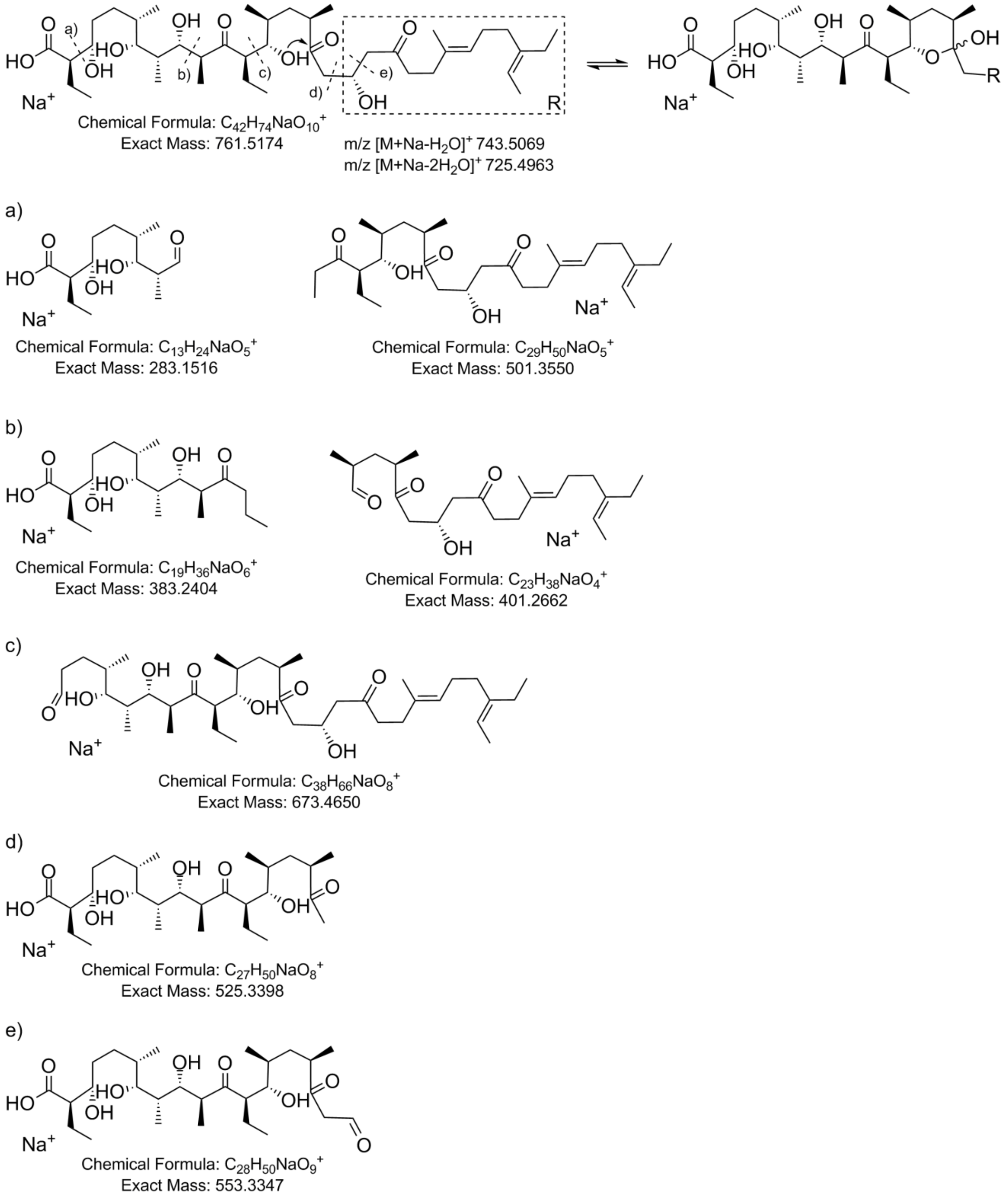

Figure S24. Proposed fragmentation pathways for compound **2**. All the fragmentations occur due to β-cleavage, a McLafferty-like rearrangement. The formation of the ions at m/z 283.2, 383.2, 525.3 and m/z 553.3 suggests the presence on two extra –OH group in the “left hand side” of the molecule. MS^3 fragmentation pattern of the m/z 761.5→383.2 confirms the presence of C3-OH and C7-OH in the molecule **2**.

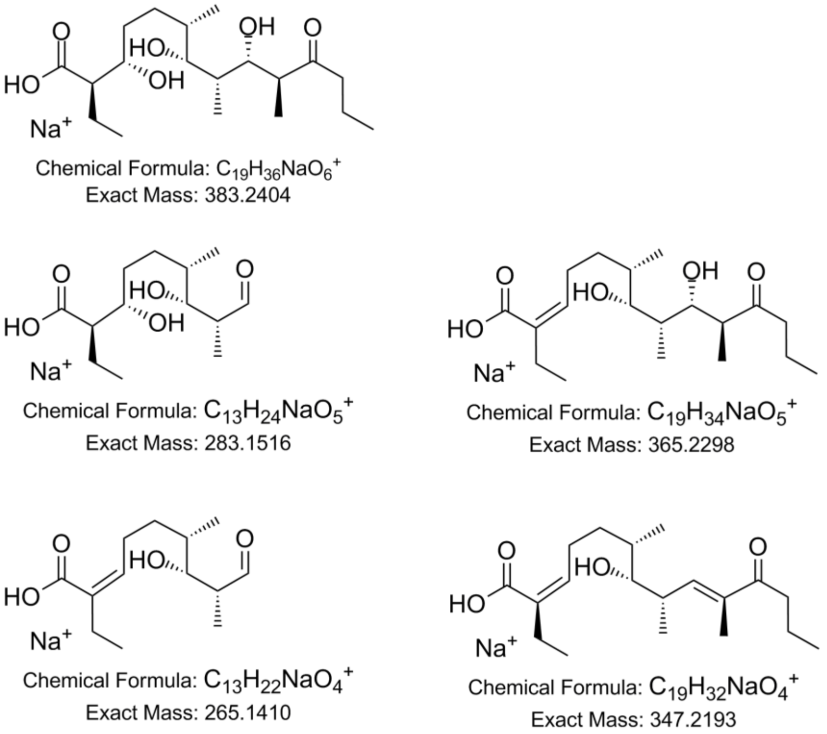

Figure S25. Proposed fragmentation pathways for compound **2**. MS^3 fragmentation pattern of the m/z 761.5→383.2 confirms the presence of C3-OH and C7-OH in the molecule **2**.

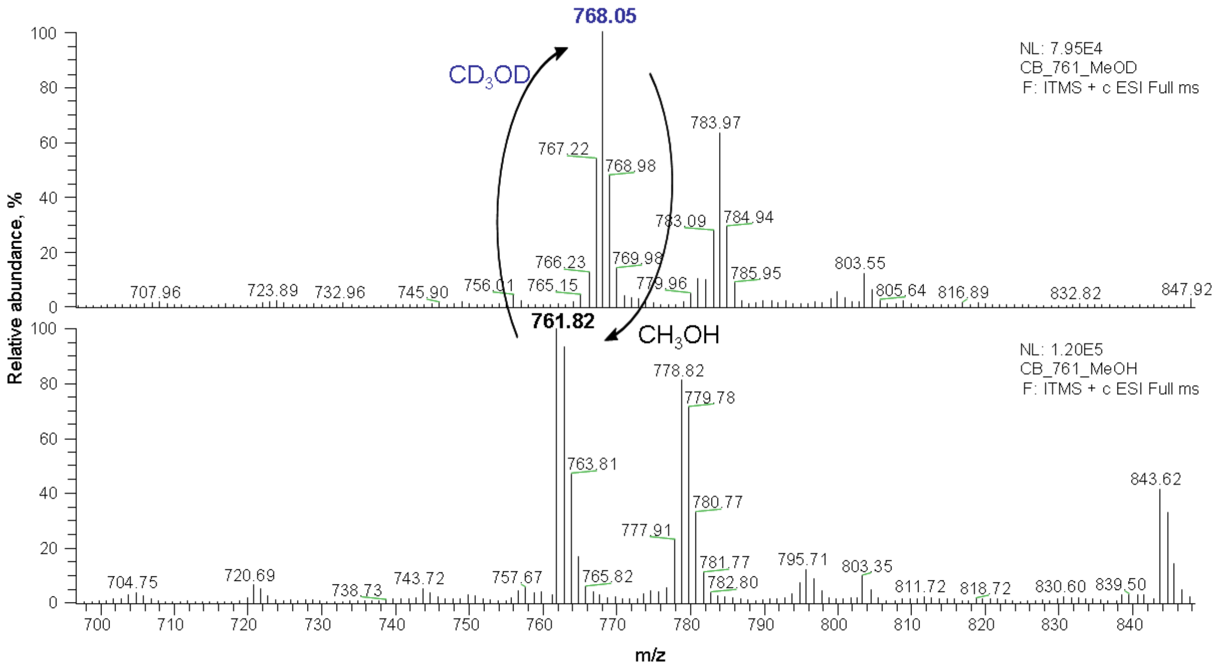

Figure S26. Comparison of MS spectrum of non-deuterated and deuterated [M+Na]$^+$ ions for compound **2**. 6 Da shift in the mass of molecular [M+Na]$^+$ ion at m/z 761.5 suggests the presence of six exchangeable protons in **2**.

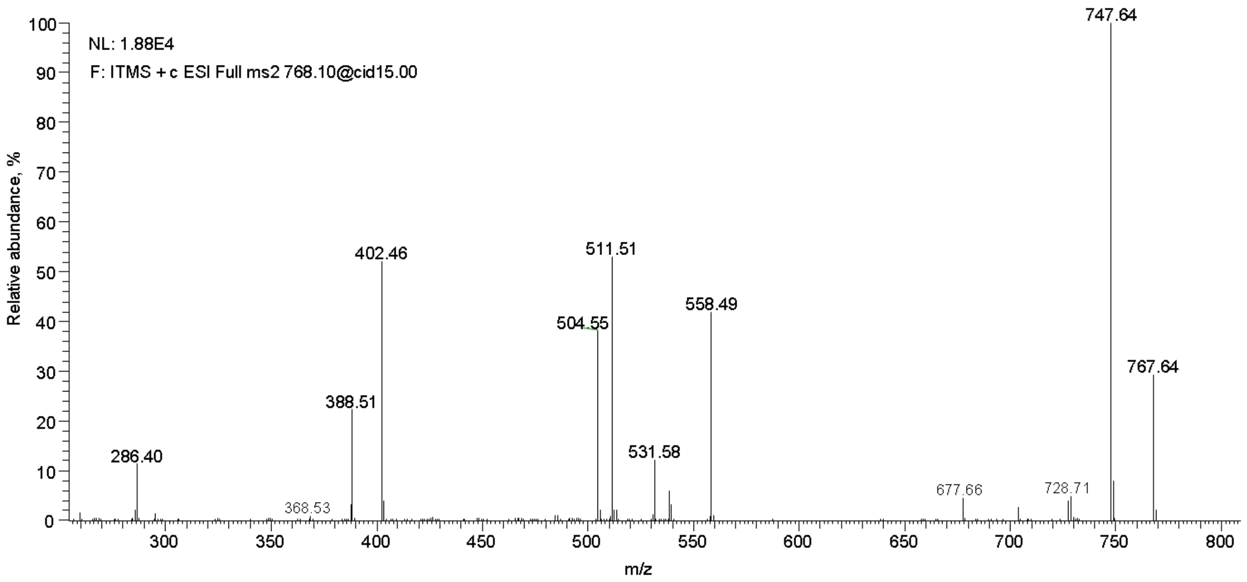

Figure S27. MS/MS spectrum of deuterated [M+Na]+ ion of compound **2**. The fragment ion at m/z 383.2 shifted to 388.5, which strongly supports the absence ofjpyran ring A in compound **2**.

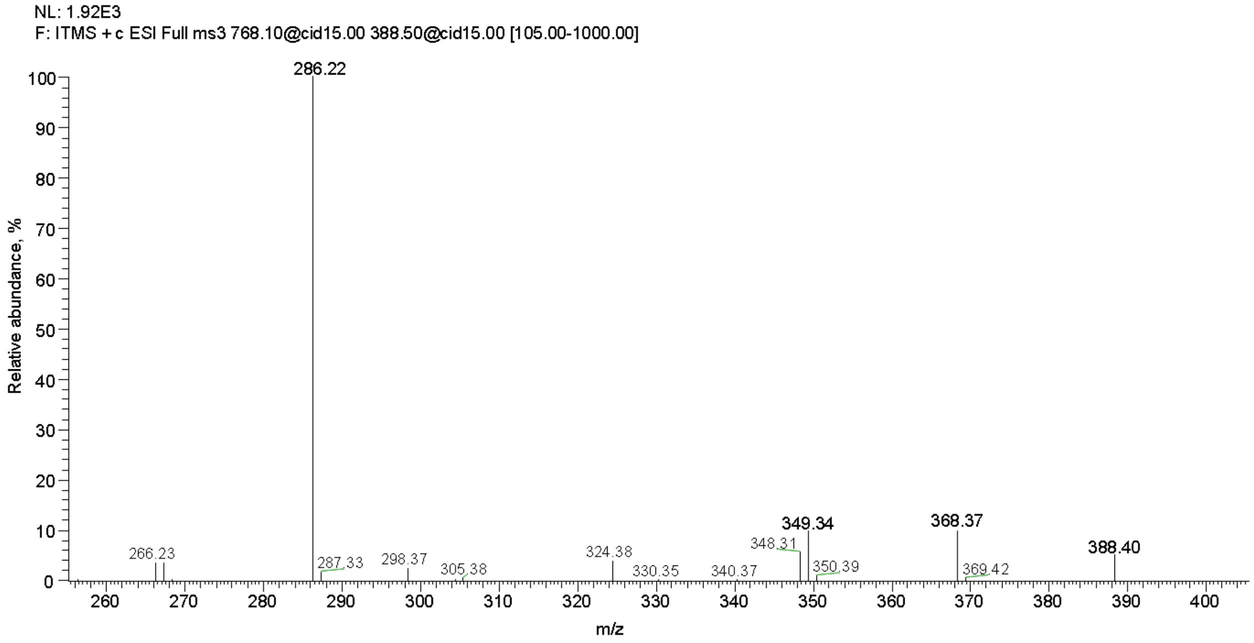

Figure S28. MS^3 spectrum of deuterated [M+Na]+ ion 767.5→388.5 of compound **2**. The fragmentation pattern confirms the absence of pyran ring A in compound **2**.

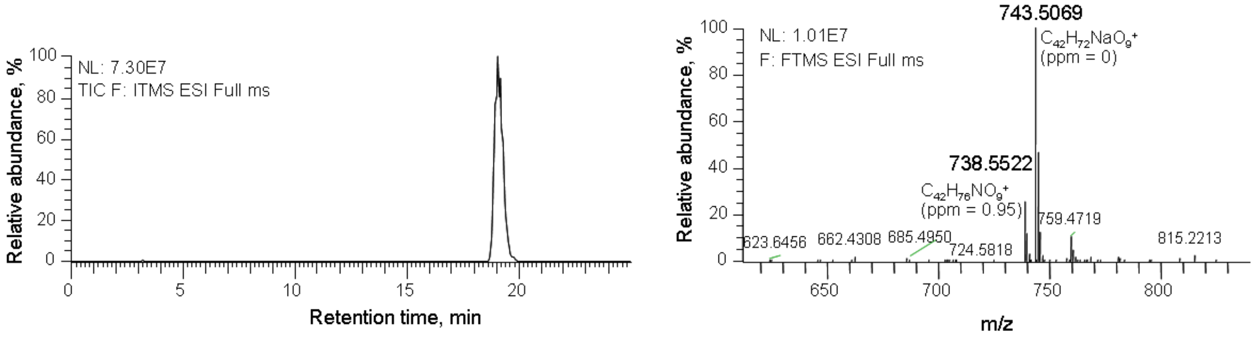

Figure S29. HPLC-HRMS analysis of purified compound **3**. HPLC method B.

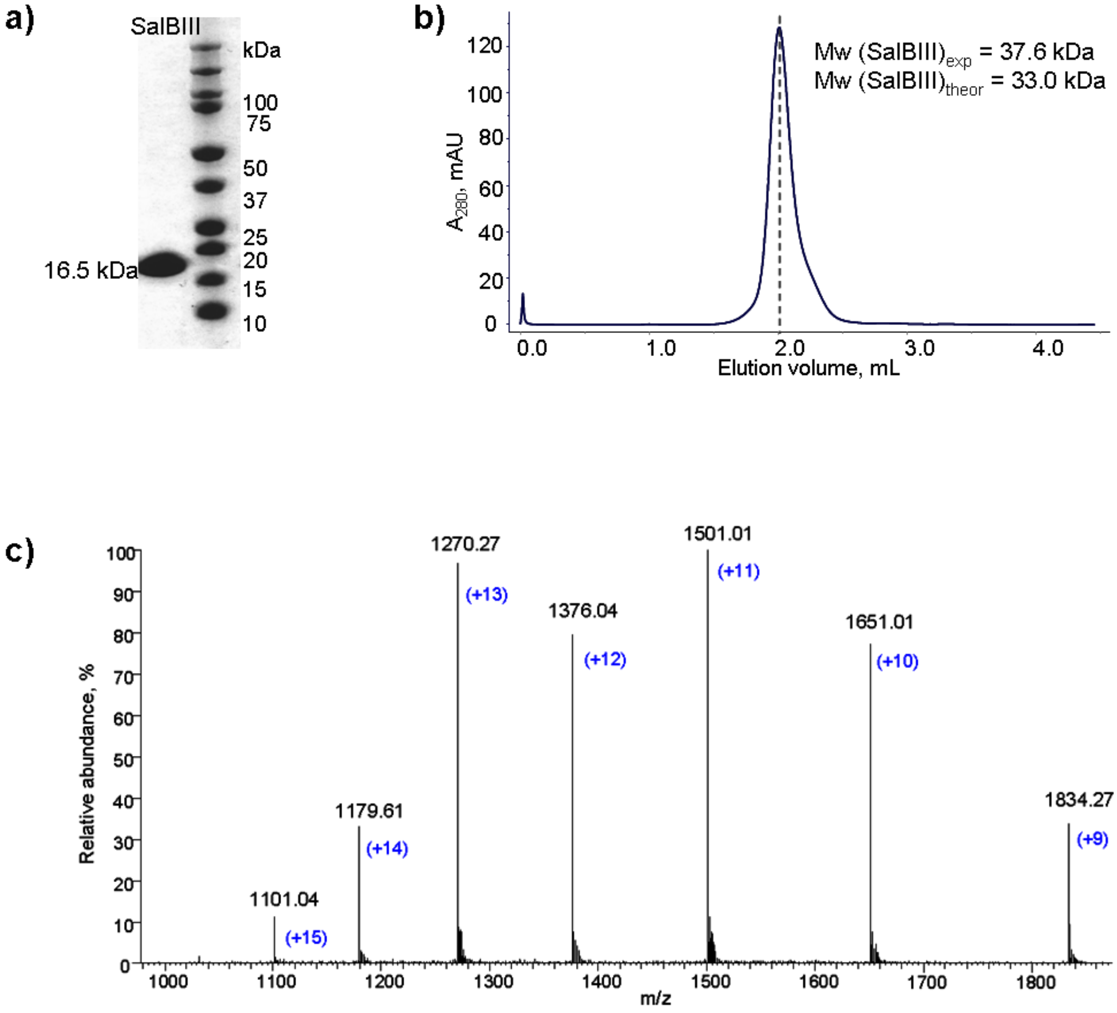

Figure S30. Analysis of the purified recombinant SalBIII protein. **a)** SDS-PAGE analysis of recombinant SalBIII protein. Precision Plus Protein^{TM} Dual Color Standards (Bio-Rad) marker was run alongside with the analysed sample. **b)** Analytical gel-filtration chromatogram of the purified SalBIII sample. **c)** MS spectrum of the purified SalBIII protein. The experimentally determined mass at 16,502.0 Da accords well with the calculated mass of 16,500.4 Da.

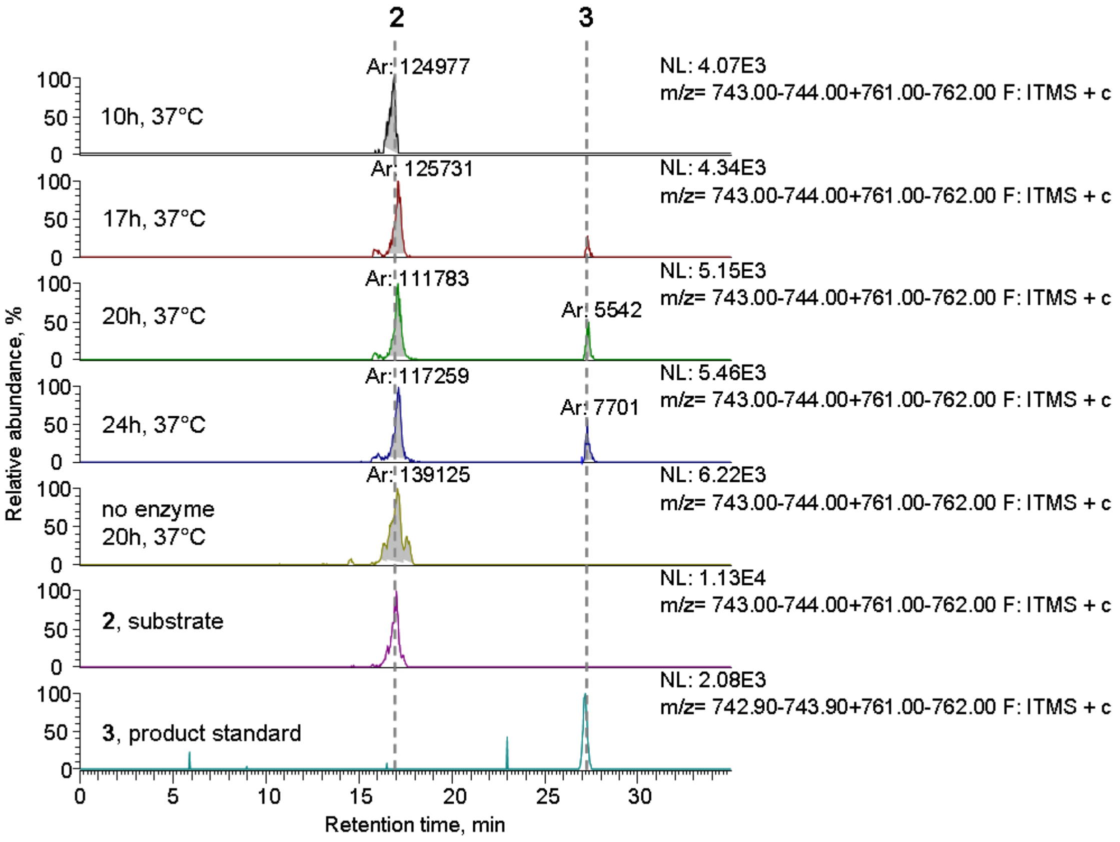

Figure S31. HPLC-MS analysis of conversion of **2** to **3** at different time points (10, 17, 20, 24 h).

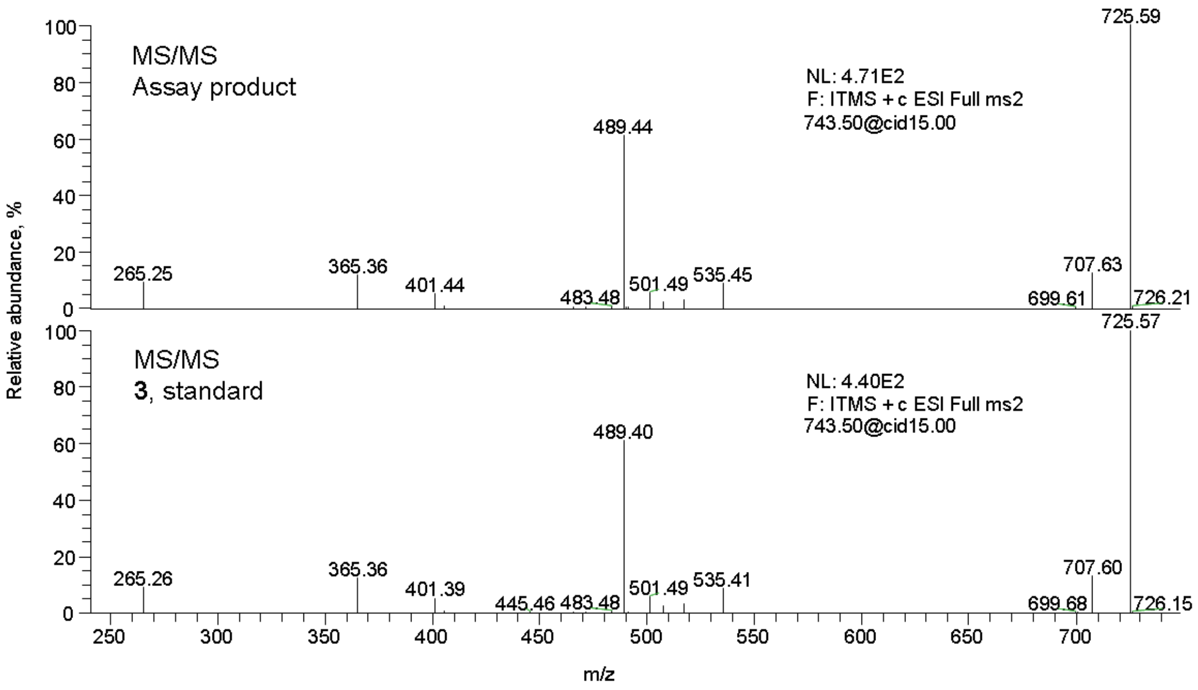

Figure S32. MS/MS analysis of the molecular ion $[M+Na]^+$ 743.5 corresponding to the assay product (top spectrum) and the **3** standard (bottom spectrum). Both molecular ions show identical fragmentation pattern.

Figure S33. ^{1}H NMR spectrum of **3** with assignments (500 MHz, CD_3CN).

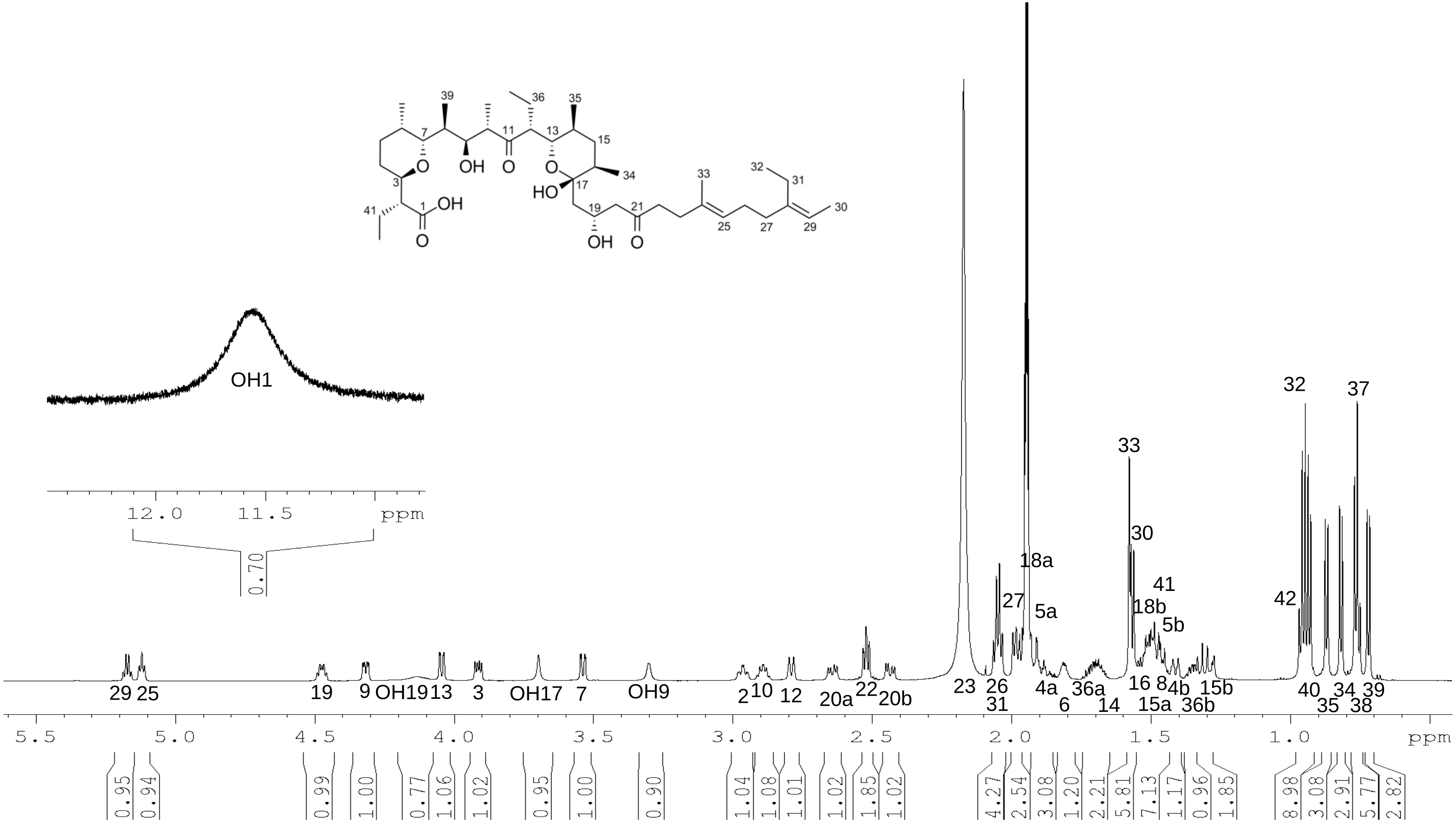

Figure S34. ^{13}C NMR spectrum of **3** with assignments (500 MHz, CD_3CN).

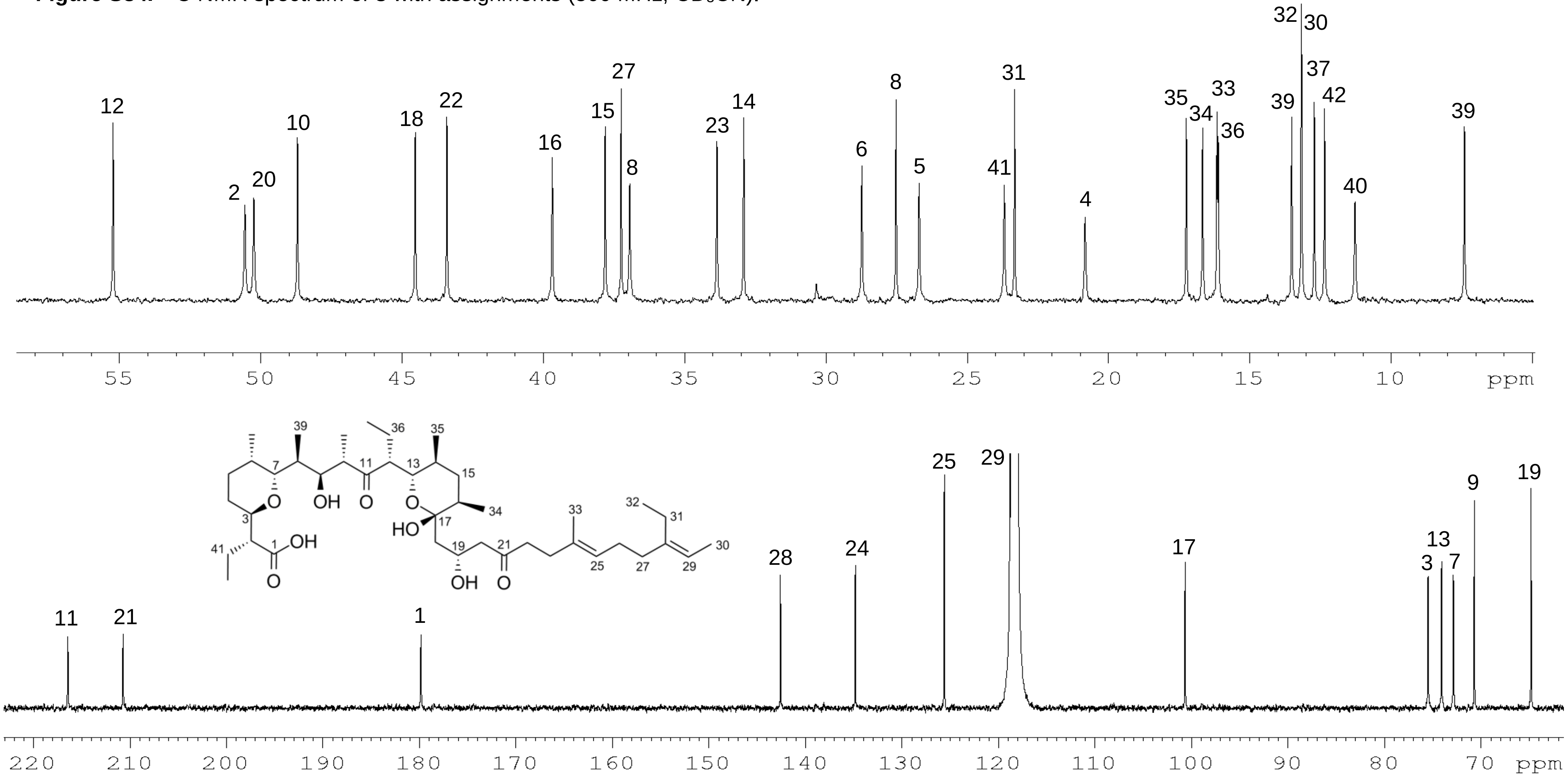

Figure S35. DEPT 135 NMR spectrum of **3** (500 MHz, CD_3CN).

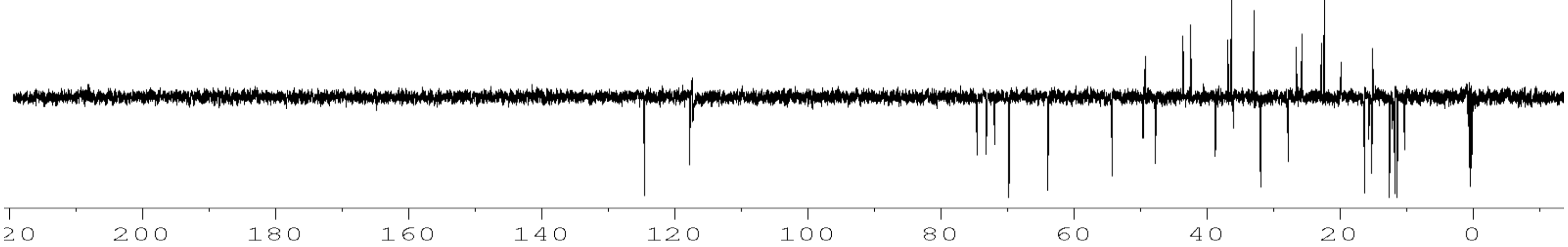

Figure S36. DEPT 90 NMR spectrum of **3** (500 MHz, CD_3CN).

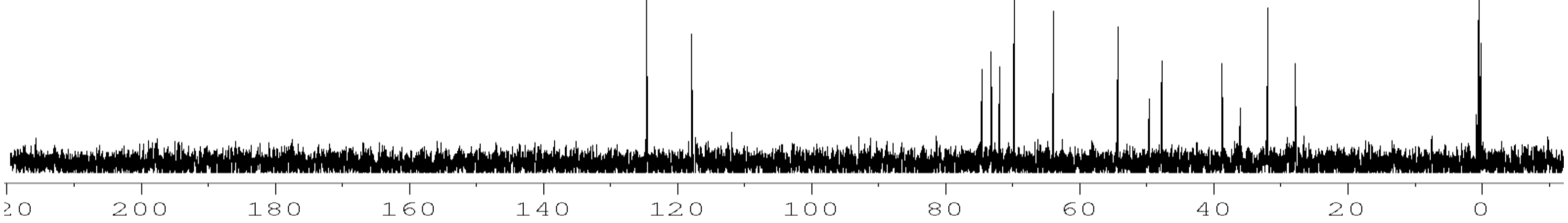

Figure S37. DEPT 45 NMR spectrum of **3** (500 MHz, CD_3CN).

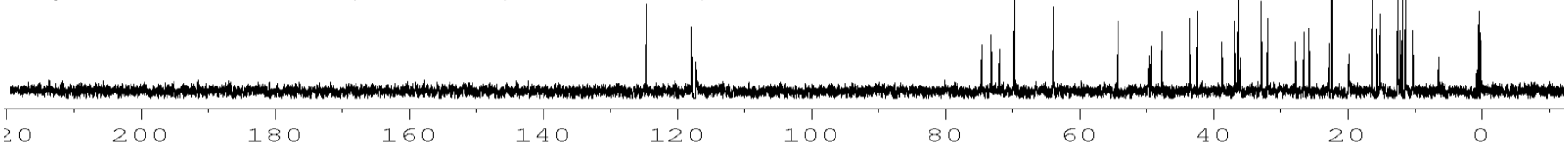

Figure S38. ^{13}C NMR spectrum of **3** (500 MHz, CD_3CN).

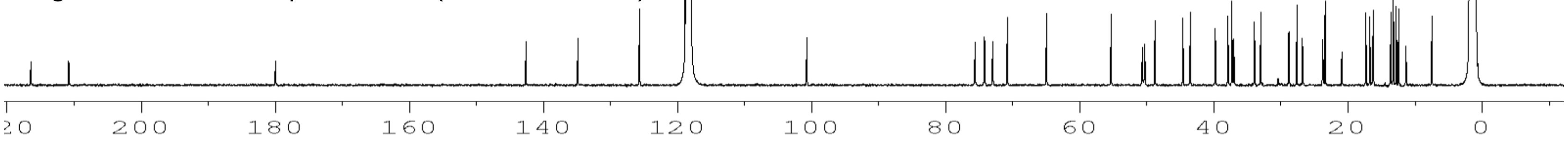

Figure S39. DQF-COSY NMR spectrum of **3** (700 MHz, CD_3CN).

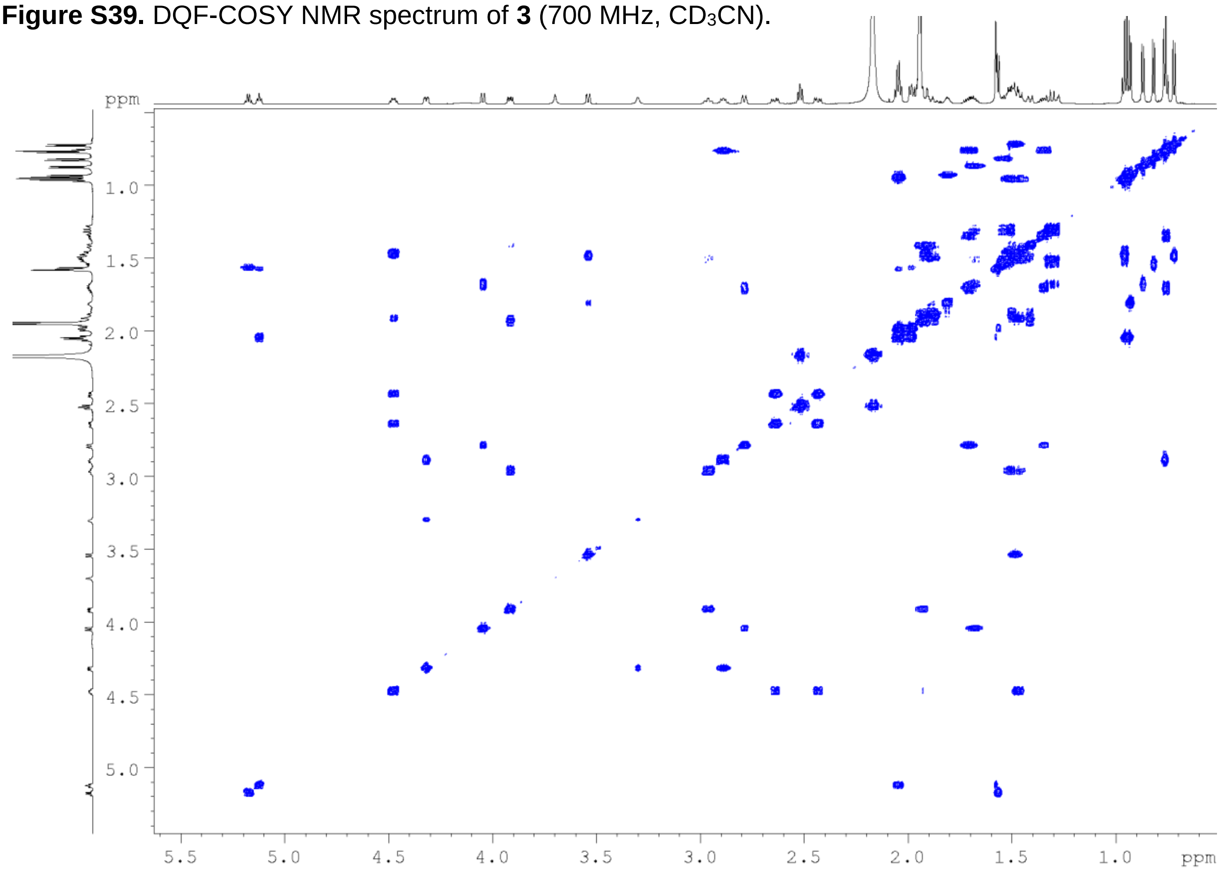

Figure S40. Edited-HSQC NMR spectrum of **3** (700 MHz, CD_3CN).

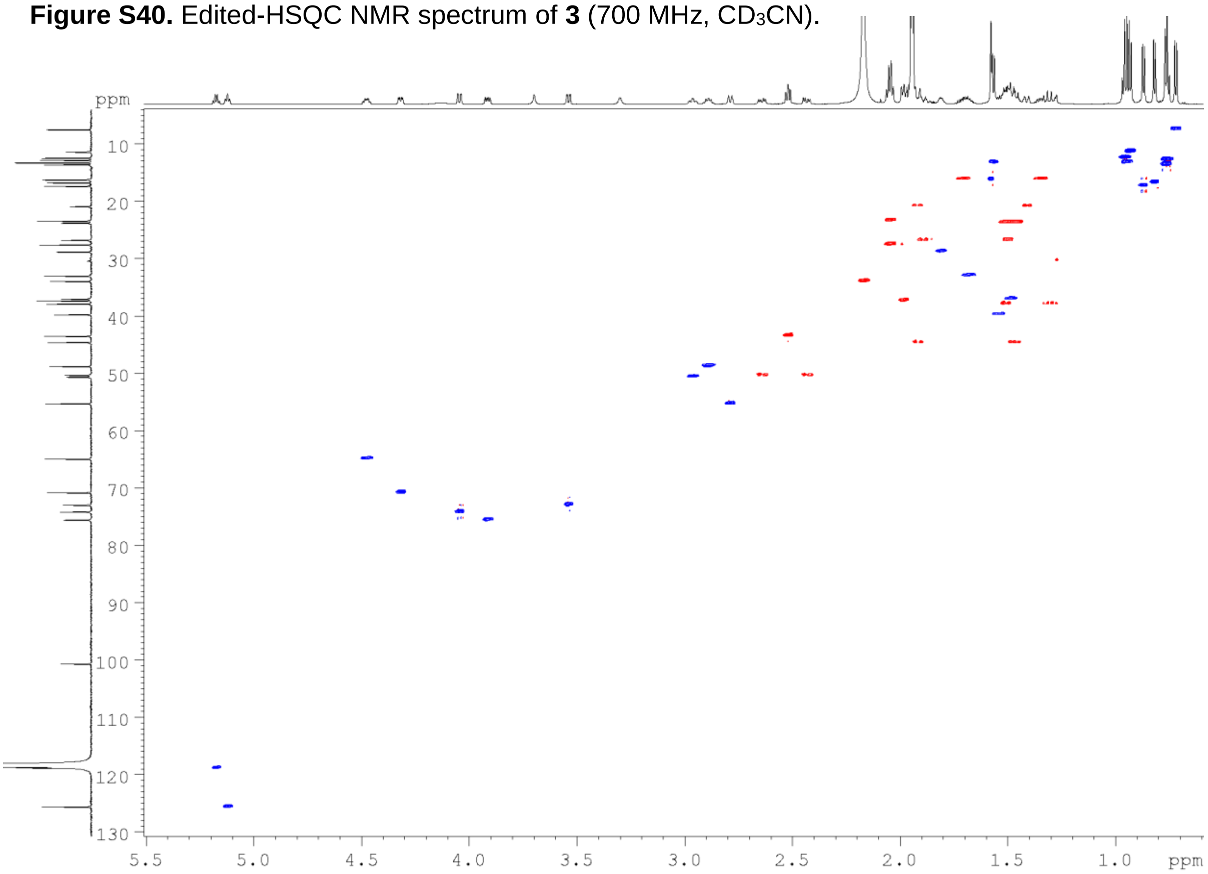

Figure S41. HMBC NMR spectrum of **3** (700 MHz, CD_3CN).

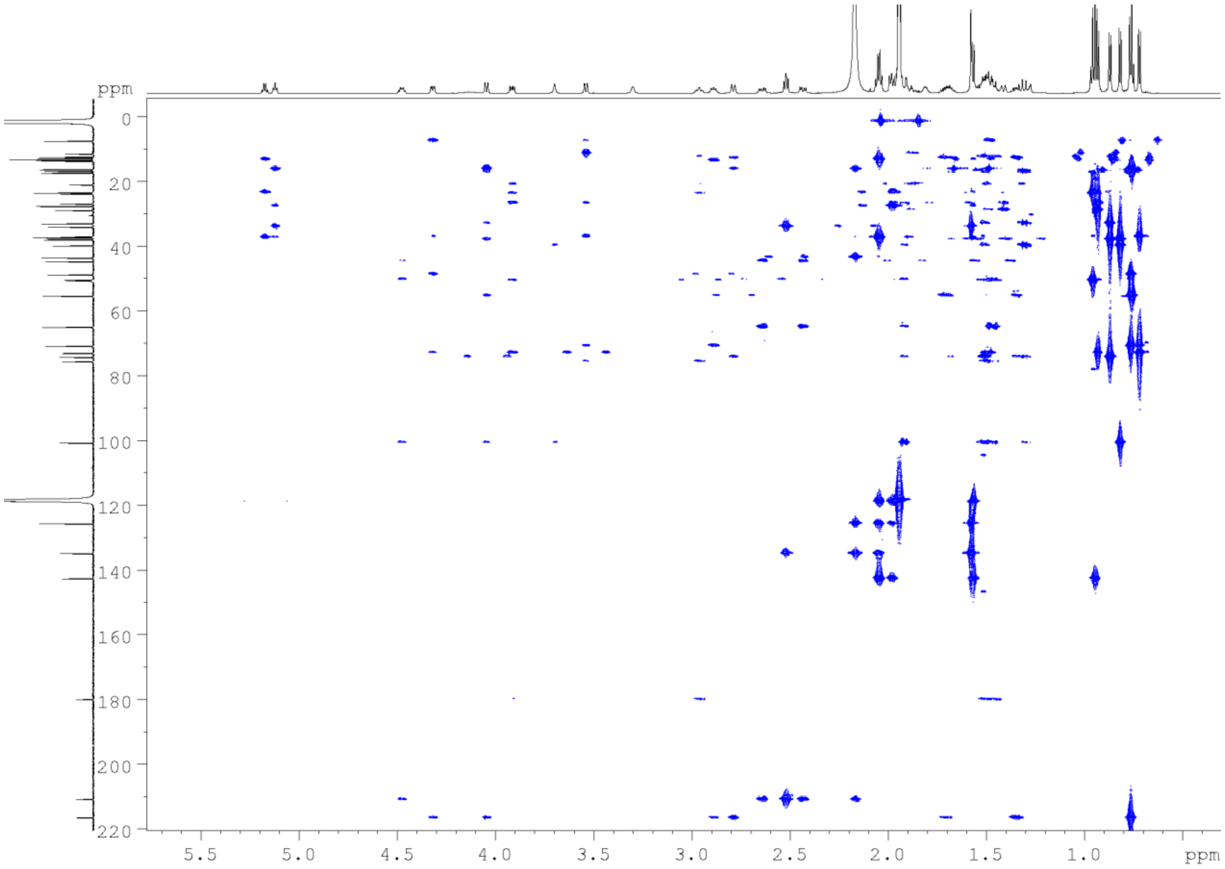

Figure S42. NOESY NMR spectrum of **3** (700 MHz, CD_3CN).

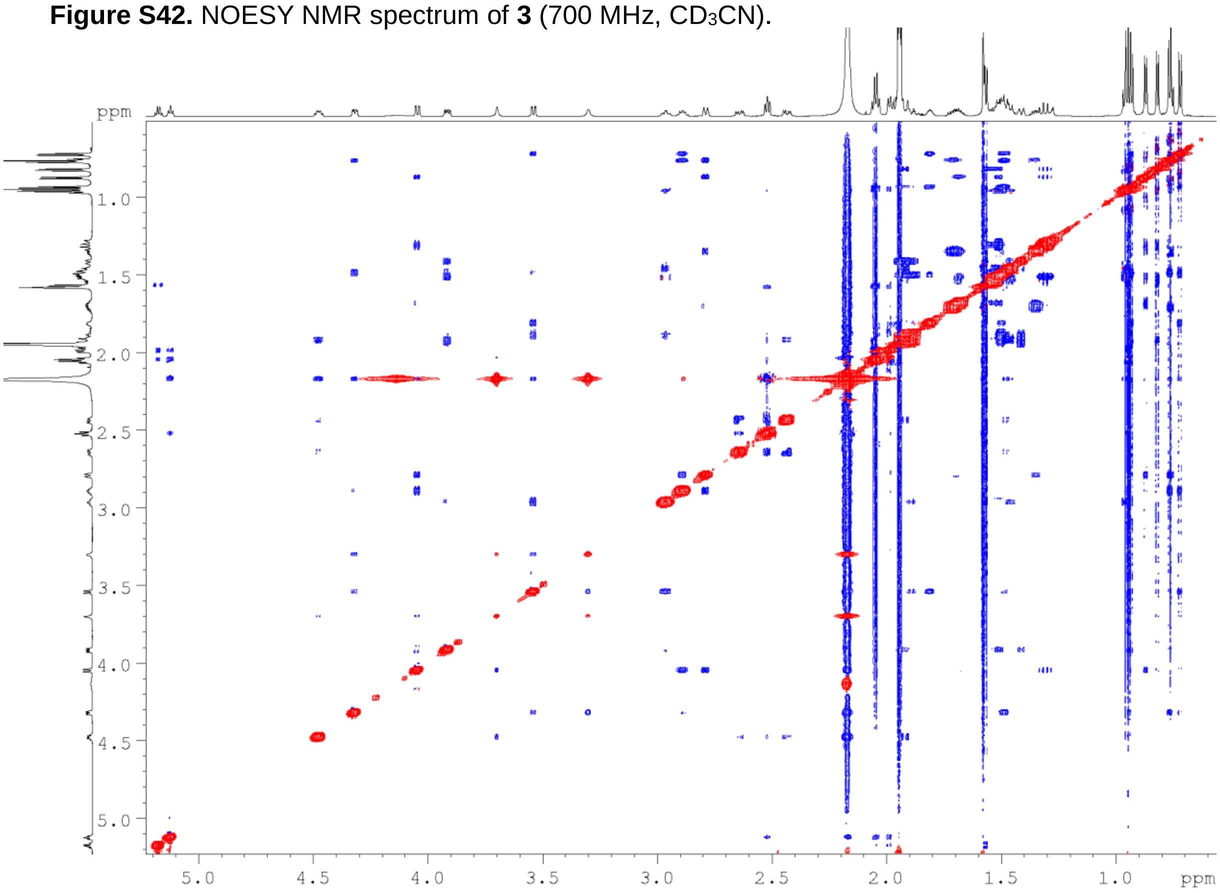

Figure S43. HSQC-TOCSY NMR spectrum of **3** (500 MHz, CD_3CN).

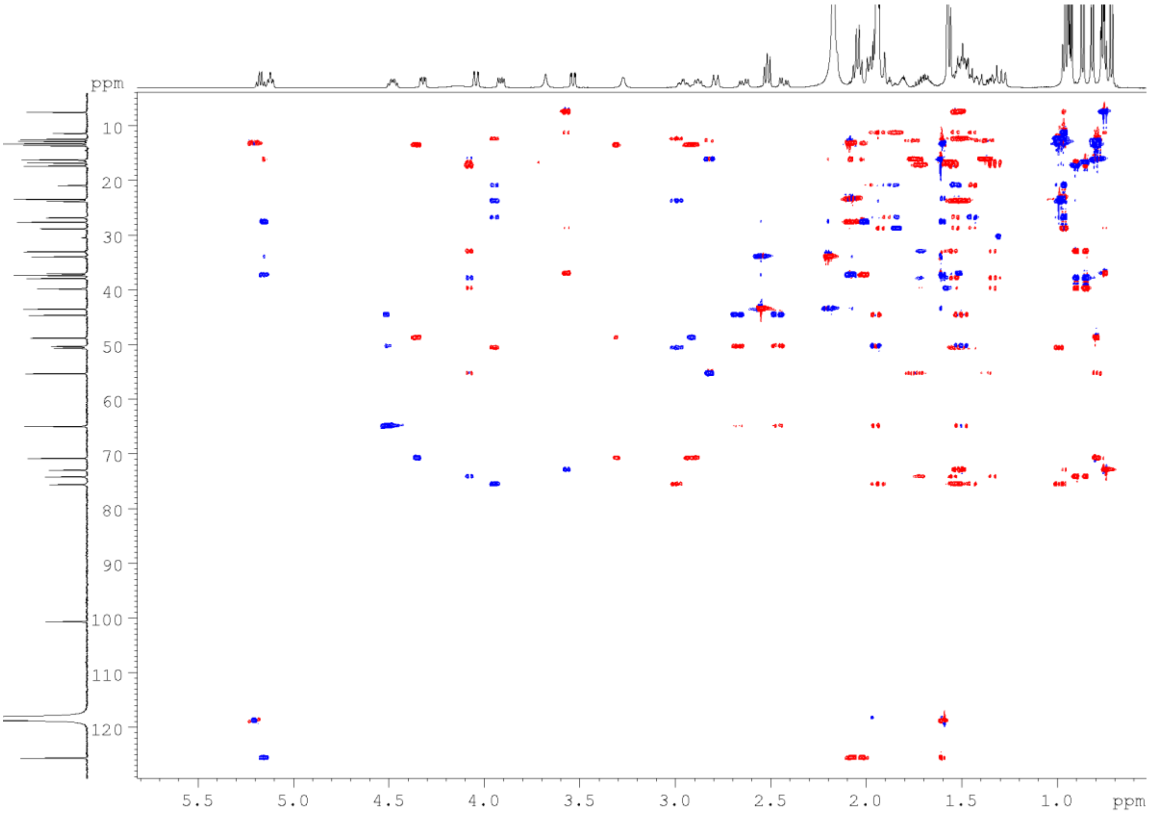

Figure S44. TOCSY NMR spectrum of **3** (500 MHz, CD_3CN).

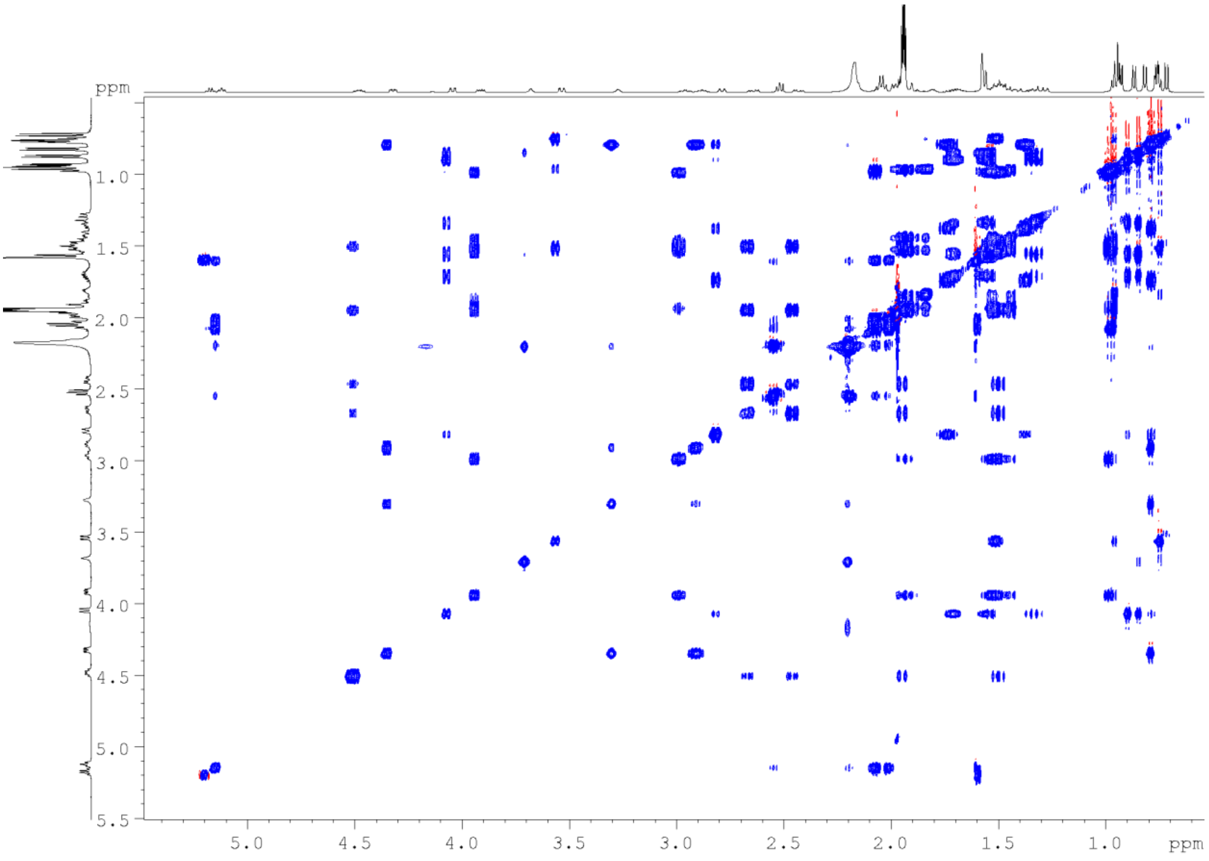

Figure S45. HSQC-HECADE NMR spectrum of **3** (500 MHz, CD_3CN).

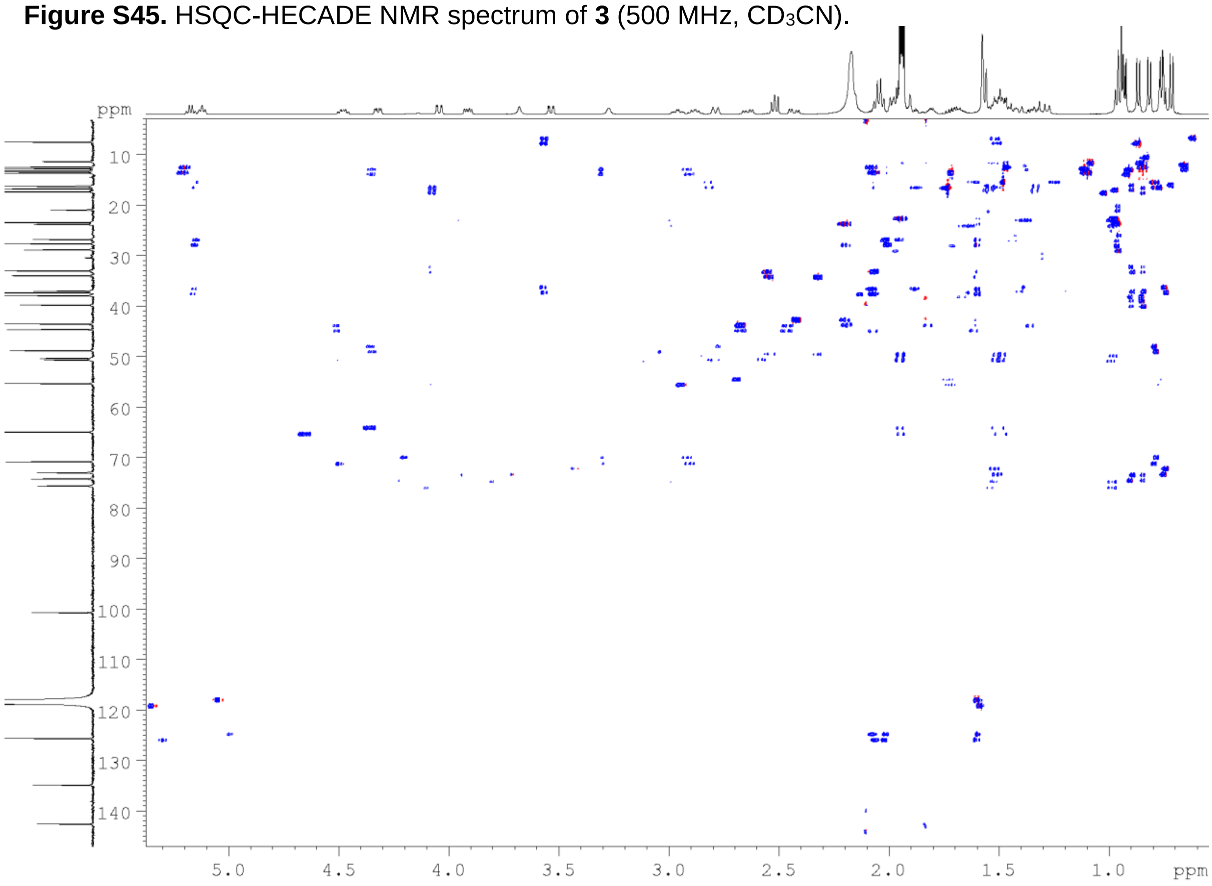

Figure S46. G-BIRD-HSQMBC NMR spectrum of **3** (500 MHz, CD_3CN).

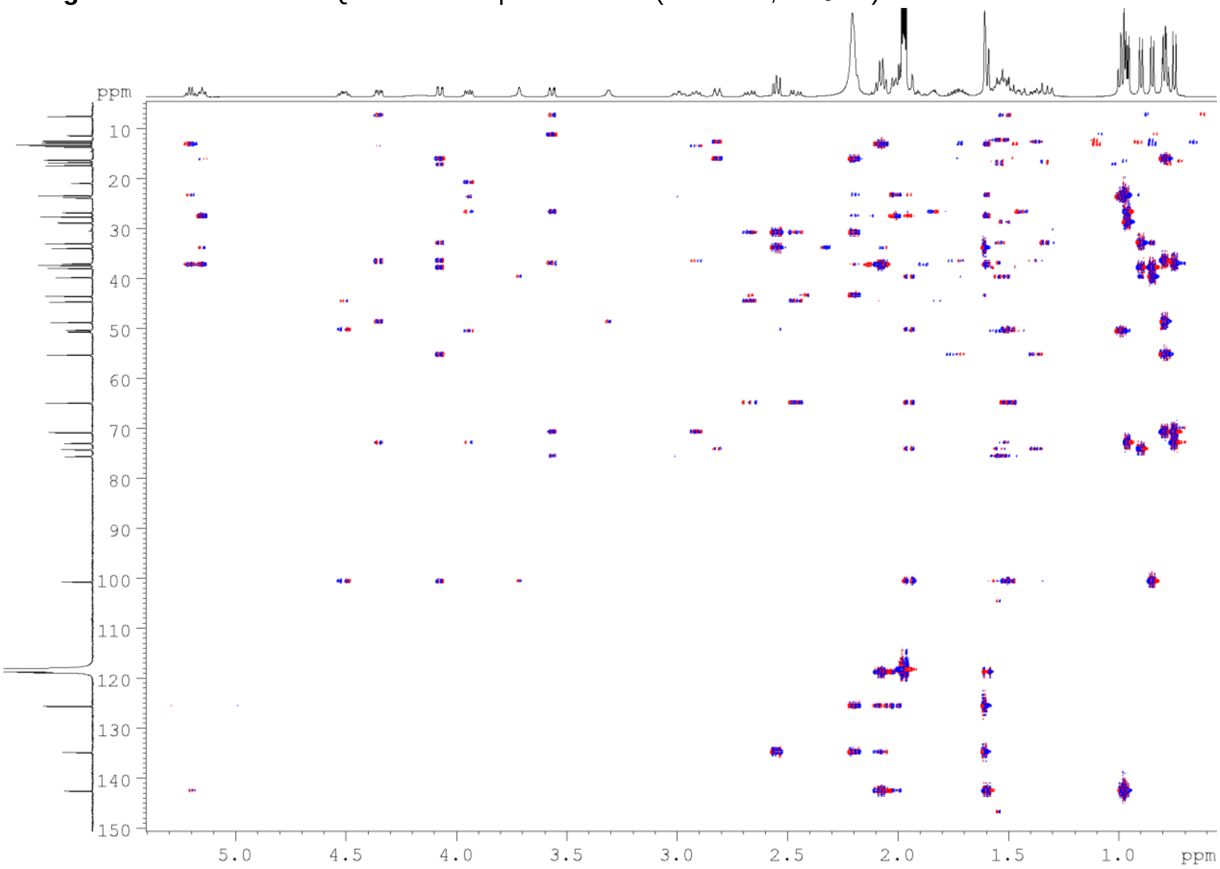

5. References

[1] T. Kieser, M. J. Bibb, M. J. Buttner, K. F. Chater, D. A. Hopwood, Practical *Streptomyces* Genetics. The John Innes Foundation, Norwich, **2000**.

[2] Y. Sun, X. He, J. Liang, X. Zhou, Z. Deng, *Appl. Microbiol. Biotechnol.* **2009**, *82*, 303–310.

[3] D. G. Gibson, L. Young, R.-Y. Chuang, J. C. Venter, C. A. Hutchison, H. O. Smith, *Nat. Methods* **2009**, *6*, 343–345.

[4] H. Luhavaya, S. R. Williams, H. Hong, L. Gonzaga de Oliveira, P. F. Leadlay, *ChemBioChem* **2014**, *15*, 2081–2085.

[5] C. J. Wilkinson, Z. A. Hughes-Thomas, C. J. Martin, I. Böhm, T. Mironenko, M. Deacon, M. Wheatcroft, G. Wirtz, J. Staunton, P. F. Leadlay, *J. Mol. Microbiol. Biotechnol.* **2002**, *4*, 417–426.

[6] M. E. Yurkovich, P. A. Tyrakis, H. Hong, Y. Sun, M. Samborskyy, K. Kamiya, P. F. Leadlay, *ChemBioChem* **2011**, *13*, 66–71.

[7] W. Kabsch, *Acta Crystallogr. D Biol. Crystallogr.* **2010**, *66*, 125–132.

[8] A. J. McCoy, *Acta Crystallogr. D Biol. Crystallogr.* **2007**, *63*, 32–41.

[9] Collaborative Computational Project, Number 4, *Acta Crystallogr. D Biol. Crystallogr.* **1994**, *50*, 760–763.

[10] K. Hotta, X. Chen, R. S. Paton, A. Minami, H. Li, K. Swaminathan, I. I. Mathews, K. Watanabe, H. Oikawa, K. N. Houk, C.-Y. Kim, *Nature* **2012**, *483*, 355–358.

[11] G. N. Murshudov, P. Skubák, A. A. Lebedev, N. S. Pannu, R. A. Steiner, R. A. Nicholls, M. D. Winn, F. Long, A. A. Vagin, *Acta Crystallogr. D Biol. Crystallogr.* **2011**, *67*, 355–367.

[12] P. V. Afonine, R. W. Grosse-Kunstleve, N. Echols, J. J. Headd, N. W. Moriarty, M. Mustyakimov, T. C. Terwilliger, A. Urzhumtsev, P. H. Zwart, P. D. Adams, *Acta Crystallogr. D Biol. Crystallogr.* **2012**, *68*, 352–367.

[13] P. D. Adams, P. V. Afonine, G. Bunkóczi, V. B. Chen, N. Echols, J. J. Headd, L.-W. Hung, S. Jain, G. J. Kapral, R. W. Grosse Kunstleve, A. J. McCoy, N. W. Moriarty, R. D. Oeffner, R. J. Read, D. C. Richardson, J. S. Richardson, T. C. Terwilliger, P. H. Zwart, *Methods* **2011**, *55*, 94–106.

[14] P. Emsley, K. Cowtan, *Acta Crystallogr. D Biol. Crystallogr.* **2004**, *60*, 2126–2132.

[15] I. W. Davis, A. Leaver-Fay, V. B. Chen, J. N. Block, G. J. Kapral, X. Wang, L. W. Murray, W. B. Arendall III, J. Snoeyink, J. S. Richardson, D. C. Richardson, *Nucl. Acids Res.* **2007**, *35*, W375–383.

[16] N. Matsumori, D. Kaneno, M. Murata, H. Nakamura, K. Tachibana, *J. Org. Chem.* **1999**, *64*, 866–876.

[17] W. Koźmiński, D. Nanz, *J. Magn. Reson.* **1997**, *124*, 383–392.

[18] R. T. Williamson, B. L. Márquez, W. H. Gerwick, K. E. Kövér, *Magn. Reson. Chem.* **2000**, *38*, 265–273.

[19] K. A. Datsenko, B. L. Wanner, *Proc. Natl. Acad. Sci. USA* **2000**, *97*, 6640–6645.

[20] L. Smith, H. Hong, J. B. Spencer, P. F. Leadlay, *Chembiochem* **2008**, *9*, 2967–2975.

[21] Y. Sun, X. Zhou, H. Dong, G. Tu, M. Wang, B. Wang, Z. Deng, *Chem. Biol.* **2003**, *10*, 431–441.

[22] A. R. Gallimore, C. B. W. Stark, A. Bhatt, B. M. Harvey, Y. Demydchuk, V. Bolanos-Garcia, D. J. Fowler, J. Staunton, P. F. Leadlay, J. B. Spencer, *Chem. Biol.* **2006**, *13*, 453–460.

[23] B. M. Harvey, T. Mironenko, Y. Sun, H. Hong, Z. Deng, P. F. Leadlay, K. J. Weissman, S. F. Haydock, *Chem. Biol.* **2007**, *14*, 703–714.

[24] Y. Demydchuk, Y. Sun, H. Hong, J. Staunton, J. B. Spencer, P. F. Leadlay, *Chembiochem* **2008**, *9*, 1136–1145.

[25] M. Arand, B. M. Hallberg, J. Zou, T. Bergfors, F. Oesch, M. J. van der Werf, J. A. M. de Bont, T. A. Jones, S. L. Mowbray, *EMBO J.* **2003**, *22*, 2583–2592.

[26] F. Corpet, *Nucl. Acids Res.* **1988**, *16*, 10881–10890.

[27] S. Guindon, J.-F. Dufayard, V. Lefort, M. Anisimova, W. Hordijk, O. Gascuel, *Syst. Biol.* **2010**, *59*, 307–321.

[28] C. Li, K. E. Roege, W. L. Kelly, *Chembiochem* **2009**, *10*, 1064–1072.

[29] C. Jiang, Z. Qi, Q. Kang, J. Liu, M. Jiang, L. Bai, *Angew. Chem. Int. Ed.* **2015**, *54*, 9097–9100; *Angew. Chem.* **2015**, *127*, 9225–9228.

6. Main Text Complete References

[6a] P. F. Leadlay, J. Staunton, M. Oliynyk, C. Bisang, J. Cortés, E. Frost, Z. A. Hughes-Thomas, S. G. Kendrew, J. B. Lester, P. F. Long, H. A. I. MacArthur, E. L. McCormick, Z. Oliynyk, C. B. W. Stark, C. J. Wilkinson, *J. Ind. Microbiol. Biotechnol.* 2001, *27*, 360–367.

[10e] K. Hotta, X. Chen, R. S. Paton, A. Minami, H. Li, K. Swaminathan, I. I. Mathews, K. Watanabe, H. Oikawa, K. N. Houk, C.-Y. Kim, *Nature* **2012**, *483*, 355–358.
